# Integrative genetic and expression profiling prioritizes *LIPA* in mononuclear phagocytes as a candidate regulator of carotid plaque

**DOI:** 10.3389/fimmu.2026.1861490

**Published:** 2026-06-19

**Authors:** Zhuyuan Yu, Shuhua Mao, Ying Song, Xiangyuan Meng, Jianing Yu, Ziyu Zong, Tianlin Gao, Hao Chen

**Affiliations:** 1 Department of Neurovascular Surgery, First Hospital of Jilin University, Changchun, China; 2 School of Public Health, Jilin University, Changchun, China; 3 School of Public Health, Qingdao University, Qingdao, China

**Keywords:** carotid plaque, *LIPA*, mononuclear phagocytes, GWAS, mendelian randomization

## Abstract

**Background:**

Atherosclerosis shows vascular bed specificity, yet research has focused on coronary arteries, leaving the causal genetics and immune cell-specific mechanisms of carotid plaque (CP) unexplored. This study aims to unbiasedly identify CP candidate genes and evaluate their therapeutic potential.

**Methods:**

Two-sample MR analysis was performed using data from 14 peripheral blood immune cell types and two GWAS datasets for carotid plaque, followed by colocalization analysis to prioritize causal genes. A human carotid plaque single-cell RNA-seq dataset was then integrated to examine cellular expression patterns, cell-cell communication, and functional enrichment. *In vitro* experiments using THP-1-derived macrophages and Jurkat T cells were conducted to validate the function of the key gene. Molecular docking and PheWAS were employed to evaluate its translational potential and possible pleiotropic associations.

**Results:**

22 candidate genes for CP were identified. *LIPA*_Mono_C showed a consistent causal association with increased CP risk in two independent GWAS datasets. Single-cell RNA sequencing confirmed that *LIPA* was highly expressed in mononuclear phagocytes of CP tissues, and CellChat analysis suggested a potential SPP1-CD44-mediated interaction between *LIPA*-expressing mononuclear phagocytes and T cells. In silico knockout of *LIPA* revealed significant enrichment of the complement and coagulation cascades. *In vitro* experiments verified that oxidized low-density lipoprotein upregulated *LIPA* in macrophages in a time- and dose-dependent manner. *LIPA* knockdown alleviated lipid accumulation, suppressed complement activation, attenuated SPP1 expression, and reduced SPP1/CD44-associated T-cell functional readouts *in vitro*. Additionally, dorsomorphin reduced oxLDL-induced *LIPA* upregulation and lipid accumulation in macrophages.

**Conclusion:**

Our integrative genetic and transcriptomic approach prioritizes *LIPA* as a mononuclear phagocyte-enriched candidate gene for CP. Functional and computational analyses suggest that *LIPA* may be associated with lipid accumulation, complement-related activation, and SPP1-CD44-associated immune regulation *in vitro*. These findings provide preliminary evidence supporting the translational relevance of *LIPA*-related modulation, pending further validation.

## 1 Introduction

Plaque in the carotid artery (CP), the typical focal manifestation of atherosclerosis, is a leading cause of ischemic stroke (1). Beyond cerebrovascular events, accumulating evidence links CP to chronic cerebral hypoperfusion, cognitive decline, and vascular dementia, imposing a substantial socioeconomic burden globally (2). Epidemiological data indicate that the detection rate of CP in adults over 50 years ranges from 30% to 50%, increasing with age (3–5). Despite advances in diagnostic and medical interventions, the precise identification and targeted intervention of vulnerable CP remain major clinical challenges. Current clinical management of CP primarily relies on lipid-lowering statins (6) and surgical revascularization (7), with a notable lack of precision therapies targeting the plaque immune microenvironment. A key reason for this translational gap is not only our insufficient understanding of the local pathological processes in CP but also the absence of causal genetic evidence directly linking gene dysregulation in specific immune cells to CP risk.

Both innate and adaptive immune cells drive the pathological process from plaque formation to rupture (8). Within the CP immune microenvironment, various immune cells coexist. Mononuclear phagocytes—particularly macrophages—are the most abundant population; upon taking up oxLDL (oxidized low-density lipoprotein), they undergo foam cell transformation, thereby generating the plaque core and fueling local inflammatory cascades (9). T cells (including CD4^+^ and CD8^+^ subsets) modulate immune responses by secreting various cytokines (8); B cells, natural killer cells, and dendritic cells have also been implicated in plaque inflammation regulation (10–12). Notably, among these immune cells, mononuclear phagocytes serve as a critical link between lipid metabolism dysregulation and the amplification of inflammatory responses (9). Their pathogenic role involves not only increased lipid uptake but also the dysregulation of intracellular cholesteryl ester hydrolysis, free cholesterol clearance, and fatty acid metabolism. Abnormalities in these processes can further promote foam cell formation, inflammatory polarization, and plaque instability (13). Therefore, screening for key regulatory molecules from the perspective of aberrant lipid handling in mononuclear phagocytes may help elucidate important mechanisms underlying CP development. Despite these advances, a critical knowledge gap remains: it is unclear whether immune cell-specific gene regulation contributes causally to carotid plaque risk, or merely reflects secondary transcriptional changes in response to plaque inflammation. Furthermore, there is a lack of systematic evaluation of the druggability and translational potential of cell-type-specific candidate genes for carotid plaque, which hinders the development of precision immunotherapies targeting the plaque microenvironment.

Recent progress in scRNA-seq and sc-eQTLs allows for cell type-specific mapping of genetic variants to gene expression (14). Using Mendelian randomization (MR) with genetic variants as tools to determine causal links, eQTL-based MR analysis evaluates how gene expression changes in specific cells relate to disease risk, reducing confounding factors and reverse causation (15–17). This strategy is superior to traditional observational studies as it allows for the unbiased screening of causal genes in complex diseases without prespecified targets. Additionally, scRNA-seq can resolve cellular heterogeneity within the plaque microenvironment, and tools such as scTenifoldKnk enable in silico gene knockout at single-cell resolution to predict downstream regulatory networks of target genes, providing a basis for subsequent mechanistic analyses (18).

This study adopted an integrated genetic and single-cell transcriptomic strategy. Specifically, Mendelian randomization, together with colocalization analyses, was performed on eQTL datasets spanning 14 peripheral immune cell subsets to pinpoint cell-type-specific genes linked to carotid plaque. We subsequently confirmed the expression profiles of these candidate genes using a human carotid plaque single-cell RNA-seq dataset, examining their expression patterns and candidate functional associations within plaque immune cell populations. We further explore preliminary druggability and phenome-wide pleiotropic associations of the lead target gene. This study aims to establish a foundation for identifying key molecules involved in carotid plaque pathogenesis and for assessing their translational potential from the perspective of immune cell genetic regulation. To functionally corroborate our multi−omics findings and provide preliminary evidence for translational relevance, we additionally conducted a series of *in vitro* experiments using THP−1−derived macrophages and Jurkat T cells. These experiments encompassed the characterization of *LIPA* expression dynamics under oxLDL stimulation, assessment of the impact of *LIPA* knockdown on lipid accumulation and complement activation, examination of SPP1-CD44-associated macrophage–T cell functional interactions, and pharmacological inhibition of *LIPA* with dorsomorphin.

## 2 Materials and methods

### 2.1 Mendelian randomization analysis

#### 2.1.1 Exposure and outcome data

The corresponding cis-eQTL data were derived from the OneK1K cohort (https://onek1k.org/) (**Supplementary Table 1**). This cohort includes single-cell sequencing data of PBMCs from 982 healthy Europeans, with cis-eQTL data for 16,597 genes across 14 cell types (19).

The corresponding GWAS summary statistics were retrieved from the GWAS Catalog database (**Supplementary Table 1**), including two independent datasets: GCST007435 (sample size = 4,843 cases) and GCST90503076 (sample size = 26,807 cases). The final access date for the above data was October 20, 2025.

#### 2.1.2 Instrument variables selection

The screening criteria for IVs were set as follows: *P* < 0.005, linkage disequilibrium coefficient *r*² < 0.01, and linkage disequilibrium block size of 5,000 kb, to ensure the independence of individual single-nucleotide polymorphism (SNP) instruments. Meanwhile, instrumental variables with minor allele frequency (MAF) > 0.01 were selected. To reduce potential pleiotropy, SNPs associated with prespecified confounders and/or the outcome were screened and excluded using the GWAS Catalog database (20) (**Supplementary Table 2**). The screened SNPs were extracted from the GWAS summary statistics for CP, excluding palindromic SNPs and weak instruments with an F-statistic below 10. MR-PRESSO was employed to remove outlier SNPs. Ultimately, all included instrumental variables were restricted to the cis-acting region ±1,000 kb of the target gene. Details of IVs and weak IVs are presented in **Supplementary Table 3** and **Supplementary Table 4**, respectively.

#### 2.1.3 Two-sample MR and sensitivity analysis

We used sc-eQTLs as instrumental variables in two-sample MR to assess the causal link between immune cell-specific gene expression and CP risk, aligning datasets by effect allele. Effect sizes were determined using the Wald ratio (single SNP) or IVW method (≥2 SNPs). We conducted three sensitivity analyses: Cochran’s Q test for heterogeneity, MR-Egger intercept test and MR-PRESSO for horizontal pleiotropy, and leave-one-out analysis for stability. All analyses were performed in R 4.1.0 (TwoSampleMR package) with α = 0.05.

To confirm MR causal direction, the MR Steiger test was used, comparing the proportion of phenotypic variance explained by instrumental variable SNPs for immune cell-specific gene expression and CP. Genetic colocalization (R package “coloc”) was performed to verify shared causal variants. We evaluated colocalization within a ±100 kb window around the gene locus using coloc. Evidence for a shared causal variant was quantified using the PPH4/(PPH3+PPH4) ratio, which reflects the posterior probability that a single genetic variant explains both gene expression and disease association signals. A threshold of >0.7 was adopted to define strong colocalization evidence, consistent with widely accepted criteria in genetic epidemiology and previous single-cell transcriptome-wide MR-colocalization studies (19, 21).

### 2.2 Single-cell RNA sequencing analysis

Single-cell RNA sequencing was performed on public dataset GSE159677 (22), including 3 atherosclerotic calcified carotid plaque (AC) samples and 3 matched adjacent normal proximal tissue (PA) samples. Analyses proceeded with decontX to remove environmental exogenous RNA contamination, followed by Seurat’s CreateSeuratObject to construct a single-cell expression matrix (22).

Post-preliminary filtration, single-cell data underwent quality control (QC) (200–4000 detected genes, mitochondrial gene proportion ≤10%). QC-passed cells were normalized (NormalizeData), batch effects removed via canonical correlation analysis (CCA), and the top 2000 highly variable genes (HVGs) selected (excluding ribosomal/mitochondrial/housekeeping genes). Dimensionality reduction (PCA, top 10 PCs) and unsupervised clustering (FindNeighbors/FindClusters, resolution=0.5) were performed, with UMAP visualization. Positive cluster markers and AC/PA differentially expressed genes (DEGs: |log_2_FC|>0.5, P<0.05) were identified. Candidate genes integrated consistent significant results from MR and scRNA-seq.

#### 2.2.1 Expression patterns of lead functional candidate gene at the single-cell level

Early multi-omics integration identified high-confidence candidate genes. Single-cell transcriptome data were used with R packages *Nebulosa* and *Seurat* to generate density heatmaps and dot plots for lead functional candidate gene expression in individual cells; cells with zero lead functional candidate gene expression were designated Negative, and high/low expression groups were stratified by the median gene expression value.

#### 2.2.2 Cell-cell communication analysis

Cell-cell communication analysis deciphered intercellular regulatory relationships mediated by signal transduction and molecular interactions. Seurat-preprocessed single-cell data with accurate cell type annotation enabled the construction of a CellChat object, and potential signaling networks were inferred using the built-in ligand-receptor database. Statistically significant ligand-receptor pairs were screened (identifyOverExpressedGenes/identifyOverExpressedInteractions), followed by sequential calculation and analysis of pathway-specific communication probabilities (computeCommunProb, filterCommunication, computeCommunProbPathway). Communication patterns were visualized (netVisual_circle, netVisual_bubble), with a focus on AC/PA group differences and mononuclear phagocyte interactions with other cell subsets.

#### 2.2.3 Differential analysis and functional enrichment of *LIPA* expression groups

To identify DEGs in mononuclear phagocytes stratified by *LIPA* expression, cells were divided into *LIPA*-expressing and *LIPA*-non-expressing groups. Differential expression analysis was conducted using Seurat’s FindAllMarkers function, identifying DEGs. Functional enrichment of these DEGs was analyzed with clusterProfiler for GO and KEGG (P < 0.05), and the top 5 enriched GO terms were visualized.

#### 2.2.4 Immune infiltration

Immune infiltration analysis characterized the carotid atherosclerosis immune microenvironment and its association with *LIPA* expression. Using dataset GSE100927 (29 atherosclerotic carotid samples, 12 normal controls), the *IOBR* package (MCPcounter database) quantified immune cell infiltration, and the *GSVA* package’s ssGSEA method calculated immune function scores for 29 gene sets (23). Wilcoxon rank-sum tests compared group differences in immune infiltration and *LIPA* expression; Spearman’s correlation analyzed the association between *LIPA* expression and immune function scores (*P* < 0.05 for statistical significance).

#### 2.2.5 scTenifoldKnk gene knockout

To further elucidate *LIPA*’s regulatory mechanism in mononuclear phagocytes of atherosclerotic calcified carotid plaque (AC) tissues, we simulated *LIPA* in silico knockout via the *scTenifoldKnk* package using scRNA-seq data of mononuclear phagocytes from the carotid plaque (CP) group in GSE159677 (24). Genes with significantly altered expression were screened (p.adjust < 0.05); functional enrichment analysis and visualization were performed with the *enrichR* and *igraph* packages (p.adjust < 0.05), with the top 20 enriched terms displayed.

### 2.3 Drug prediction, molecular docking, and PheWAS

#### 2.3.1 Drug prediction and molecular docking

To identify potential novel drugs targeting *LIPA*, small-molecule drugs against the *LIPA* gene were screened from DrugBank and the CTD. For CTD screening, species were restricted to *Homo sapiens*, and compounds with documented LIPA-related interactions were retained for subsequent network and docking analyses. The LIPA-drug interaction network was visualized using Cytoscape software.

Molecular docking was conducted using CB-Dock2 to assess the binding affinity between screened drugs and the LIPA protein. Drug structures were sourced from PubChem, and the highest-resolution LIPA protein crystal structure was obtained from the PDB. Subsequent molecular docking and visualization were conducted using CB-Dock2.

#### 2.3.2 PheWAS analysis

PheWAS is a reverse genetics analysis method aimed at investigating which phenotypes may be associated with a given genetic variant. To further investigate whether the *LIPA* gene exerts effects on other traits, a gene-level PheWAS was performed. Phenotypic data were retrieved from the AstraZeneca PheWAS Portal database, which included 17,361 binary phenotypes and 1,419 quantitative phenotypes (17, 25, 26).

### 2.4 Cell culture and reagents

THP-1 monocytes (Procell, China) were grown in RPMI 1640 plus 10% FBS. Jurkat T cells (Procell) were cultured similarly. OxLDL was sourced from YEASEN (Shanghai, China), and dorsomorphin from Yuanye (S31490-25mg, Shanghai, China) was dissolved in DMSO to 10 mM. Recombinant human SPP1 protein and anti-human CD44 antibody were from R&D Systems (USA).

### 2.5 OxLDL treatment and siRNA transfection

In time course experiments, THP-1 macrophages were exposed to 100 μg/mL oxLDL for different times. For dose response, cells received 0, 25, 50, and 100 μg/mL oxLDL for 24 hours. For *LIPA* knockdown, macrophages were transfected with a commercial human *LIPA* pre-designed siRNA set (HY-RS07684, MedChemExpress, USA), which includes siRNAs targeting human *LIPA* and a non-targeting scrambled negative control siRNA. Transfection was performed using Lipofectamine RNAiMAX (Invitrogen, USA). Because the exact siRNA sequences are proprietary and not disclosed by the manufacturer, the unique catalog number is provided for reagent traceability.

### 2.6 RNA extraction and quantitative real-time PCR

Total RNA was isolated from THP-1 macrophages using TRIzol and reverse-transcribed with the PrimeScript RT Kit (TaKaRa). qPCR was performed on an ABI 7500 Fast System using SYBR Premix Ex Taq II (TaKaRa). Human *LIPA* qPCR Primer Pair (QH17517S, Beyotime, Shanghai, China), GAPDH forward, 5′-GGAGCGAGATCCCTCCAAAAT-3′ and reverse, 5′-GGCTGTTGTCATACTTCTCATGG-3′. Relative mRNA expression was calculated using the 2^-^ΔΔCt method with GAPDH as the internal control.

### 2.7 Western blot analysis

Protein concentrations were determined via BCA assay. 30 μg proteins were separated by 10% SDS-PAGE, transferred to PVDF membranes, blocked with 5% non-fat milk in TBST, and incubated overnight at 4°C with primary antibodies. After washing, membranes were probed with HRP-conjugated secondary antibodies, and protein bands were visualized using an ECL kit. *LIPA* (1:1000, ab154356, Abcam), SPP1 (1:1000, EPR21139-316), C1QA (1:1000, 11602-1-AP, Proteintech), C1QB (1:1000, 16919-1-AP), C1QC (1:1000, 16889-1-A), C3 (1:1000, 21337-1-A), Phospho-c-JUN (Ser73) (1:1000, Cell Signaling Technology, #3270), c-JUN (1:1000, #9165), and β-actin (1:5000, 66009-1-Ig, Proteintech).

### 2.8 Oil red O staining

THP-1 macrophages were fixed with 4% paraformaldehyde for 20 min, rinsed with PBS, and stained for 15 min with filtered Oil Red O solution. Images were acquired using an inverted microscope at 20× magnification.

### 2.9 Transwell co-culture assay and T-cell functional analysis

To evaluate *LIPA*-associated SPP1-CD44-mediated crosstalk between macrophages and T cells, a non-contact Transwell co-culture system using THP-1-derived macrophages and Jurkat T cells was established. Briefly, THP-1-derived macrophages were seeded in the lower chamber. After cell attachment, Jurkat T cells were seeded in the upper chamber containing a 0.4 μm pore-size polycarbonate membrane at a 1:1 ratio relative to macrophages. Recombinant human SPP1 protein (100 ng/mL), anti-human CD44 antibody (10 μg/mL), or their combination was added to the indicated groups. The two cell populations were co-cultured in RPMI 1640 medium supplemented with 10% fetal bovine serum for 48 h. At the end of co-culture, Jurkat T cells from the upper chamber were collected for flow cytometric analysis of PD-1 and LAG-3 expression and cell-cycle distribution, whereas co-culture supernatants were harvested for IFN-γ measurement by ELISA.

### 2.10 Statistical analysis

Data are shown as mean ± SD. Statistical comparisons were made with GraphPad Prism 9 using unpaired two-tailed *t*-tests or one-way ANOVA with Tukey’s *post hoc* test (≥ three groups). *P* < 0.05 was considered significant.

## 3 Results

### 3.1 Summary of instrument selection for immune-cell specific gene expressions

A total of 17,910 independent cis-sc-eQTLs corresponding to 16,563 genes across 14 cell types were obtained (**Supplementary Figure 1**). After matching with outcome datasets and MR-PRESSO outlier detection, 31 sc-eQTLs with F-statistics < 10 (weak IVs) were excluded. Finally, 14,634 (GCST007435) and 14,735 (GCST90503076) harmonized instrument–outcome pairs (29,369 total) were retained, corresponding to 14,083 immune cell-specific genes for MR analysis. The sequential filtering criteria and the number of instruments or gene–cell type pairs retained at each step are summarised in **Supplementary Table 5**. Here, the term ‘immune cell−specific gene’ refers to a gene–cell type pair (e.g., *LIPA*_Mono_C) evaluated in a specific immune cell type, rather than a unique gene symbol. When referring to unique genes irrespective of cell type, we explicitly use the term ‘unique genes.’

### 3.2 Identification of MR-prioritized candidate gene for carotid plaque

**Figure 1:**
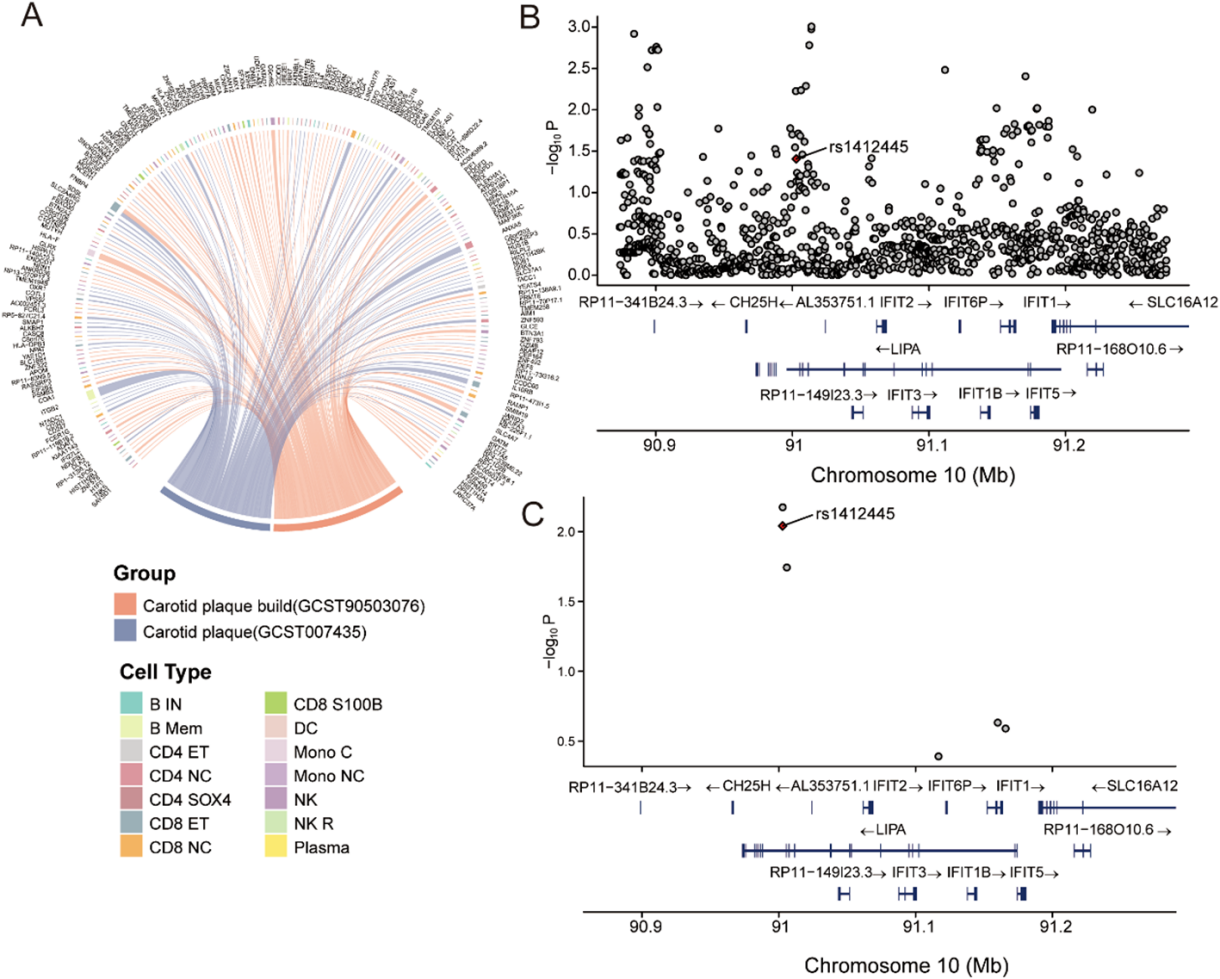
Mendelian randomization analysis identifies immune cell-specific genes causally associated with carotid plaque. **(A)** Chord diagram showing the top 200 immune cell-specific genes with nominal MR association with CP (*P* < 0.05), with each chord linking genes to their corresponding immune cell types. **(B)** Regional association plot of *LIPA*_Mono_C gene expression with CP risk in the GCST007435 dataset, with the x-axis representing chromosome 10 positions (Mb) and the y-axis representing the MR association significance; the red dot indicates the lead SNP (rs1412445) for *LIPA*_Mono_C. **(C)** Regional association plot of *LIPA*_Mono_C gene expression with CP risk in the GCST90503076 dataset, consistent with the genomic localization and association trend in the GCST007435 dataset.

MR analysis identified 1,489 immune cell-specific genes showing nominal evidence of association with CP (*P* < 0.05), including 911 genes across 14 immune cell types (**Figure 1A**; **Supplementary Figure 2**). MR signals were concentrated in CD4 NC, NK, and CD8 ET subsets (**Supplementary Figure 3**). Heterogeneity and horizontal pleiotropy analyses showed no significant heterogeneity in 86 associations with ≥2 IVs, nor horizontal pleiotropy in 6 associations with ≥3 IVs (**Supplementary Tables 6, S7**). For single-SNP instruments, heterogeneity and pleiotropy testing are not applicable; robustness was therefore assessed primarily through cross-dataset replication, Steiger directionality, and colocalization (see below).

Cross-dataset comparison identified 45 immune cell-specific genes associated with CP in both datasets (*P* < 0.05, **Supplementary Figure 4**), with 33 showing consistent causal direction (*P* < 0.05, consistent OR direction; **Supplementary Figure 5**). MR Steiger tests supported the direction from genetically predicted expression to CP for these 33 genes (**Supplementary Table 8**), and colocalization analysis further prioritized 22 genes with strong cross-dataset evidence for a shared causal variant between expression and CP signals (e.g., *LIPA*_Mono_C, *HNRNPU*_CD4_NC; **Supplementary Table 8**; **Figures 1B, C**). Collectively, these 22 high-confidence candidate genes represent high-confidence candidates supported by replication, directionality testing, and colocalization (**Table 1**). For example: *LIPA*_Mono_C was linked to increased CP risk (GCST90503076: OR=1.093, 95% CI=1.022–1.168, *P*=0.009); *HNRNPU*_CD4_ET (GCST007435: OR=0.670, 95% CI=0.495–0.905, *P*=0.009) and *VCL*_CD8_NC (GCST007435: OR=0.894, 95% CI=0.817–0.987, *P*=0.014) were associated with decreased risk. All ORs reflect genetically predicted increases in cell-type-specific gene expression (instrumented by cis-eQTLs).

**Table 1:** Association results between immune cell-specific hub gene expression and carotid plaque.

Exposure	Outcome	OR (95%CI)	*P*-value	PPH4/ (PPH3+PPH4)
*AC002451.3*_Mono_NC	Carotid plaque (GCST007435)	1.053 (1.005-1.104)	0.029	0.997
*AC002451.3*_Mono_NC	Carotid plaque build (GCST90503076)	1.057 (1.005-1.111)	0.031	1.000
*COG5*_CD8_S100B	Carotid plaque (GCST007435)	1.104 (1.007-1.210)	0.035	1.000
*COG5*_CD8_S100B	Carotid plaque build (GCST90503076)	1.107 (1.001-1.223)	0.047	1.000
*DLK2*_CD4_NC	Carotid plaque (GCST007435)	0.806 (0.657-0.990)	0.039	1.000
*DLK2*_CD4_NC	Carotid plaque build (GCST90503076)	0.761 (0.627-0.924)	0.006	1.000
*ELL2*_NK	Carotid plaque (GCST007435)	0.819 (0.692-0.969)	0.020	1.000
*ELL2*_NK	Carotid plaque build (GCST90503076)	0.793 (0.658-0.955)	0.015	1.000
*FNBP4*_CD4_NC	Carotid plaque (GCST007435)	0.928 (0.878-0.981)	0.008	1.000
*FNBP4*_CD4_NC	Carotid plaque build (GCST90503076)	0.937 (0.883-0.994)	0.030	1.000
*FNBP4*_CD8_NC	Carotid plaque (GCST007435)	0.878 (0.797-0.967)	0.008	1.000
*FNBP4*_CD8_NC	Carotid plaque build (GCST90503076)	0.892 (0.804-0.989)	0.030	1.000
*FNBP4*_CD8_S100B	Carotid plaque (GCST007435)	0.811 (0.696-0.945)	0.007	1.000
*FNBP4*_CD8_S100B	Carotid plaque build (GCST90503076)	0.815 (0.700-0.949)	0.008	1.000
*HNRNPU*_CD4_ET	Carotid plaque (GCST007435)	0.670 (0.495-0.905)	0.009	1.000
*HNRNPU*_CD4_ET	Carotid plaque build (GCST90503076)	0.751 (0.614-0.918)	0.005	1.000
*KIF2A*_CD8_NC	Carotid plaque (GCST007435)	0.873 (0.768-0.992)	0.037	1.000
*KIF2A*_CD8_NC	Carotid plaque build (GCST90503076)	0.870 (0.759-0.999)	0.048	1.000
*LIPA*_Mono_C	Carotid plaque (GCST007435)	1.069 (1.003-1.138)	0.039	1.000
*LIPA*_Mono_C	Carotid plaque build (GCST90503076)	1.093 (1.022-1.168)	0.009	1.000
*ORMDL3*_CD8_S100B	Carotid plaque (GCST007435)	1.106 (1.023-1.197)	0.012	1.000
*ORMDL3*_CD8_S100B	Carotid plaque build (GCST90503076)	1.107 (1.018-1.203)	0.017	1.000
*ORMDL3*_NK	Carotid plaque (GCST007435)	1.042 (1.002-1.084)	0.040	1.000
*ORMDL3*_NK	Carotid plaque build (GCST90503076)	1.047 (1.004-1.091)	0.030	1.000
*PLEKHA1*_CD4_NC	Carotid plaque (GCST007435)	1.055 (1.000-1.113)	0.049	1.000
*PLEKHA1*_CD4_NC	Carotid plaque build (GCST90503076)	1.061 (1.003-1.122)	0.040	1.000
*PLEKHA1*_CD8_NC	Carotid plaque (GCST007435)	1.089 (1.000-1.185)	0.049	1.000
*PLEKHA1*_CD8_NC	Carotid plaque build (GCST90503076)	1.098 (1.004-1.200)	0.040	1.000
*RP1-313I6.12*_B_Mem	Carotid plaque (GCST007435)	1.255 (1.043-1.510)	0.016	1.000
*RP1-313I6.12*_B_Mem	Carotid plaque build (GCST90503076)	1.259 (1.043-1.520)	0.016	1.000
*RP11-179B2.2*_CD4_NC	Carotid plaque (GCST007435)	1.121 (1.001-1.255)	0.048	1.000
*RP11-179B2.2*_CD4_NC	Carotid plaque build (GCST90503076)	1.147 (1.015-1.296)	0.028	1.000
*RP11-386I14.4*_NK	Carotid plaque (GCST007435)	1.175 (1.019-1.356)	0.027	1.000
*RP11-386I14.4*_NK	Carotid plaque build (GCST90503076)	1.150 (1.002-1.319)	0.046	1.000
*RP11-452H21.4*_B_Mem	Carotid plaque (GCST007435)	0.816 (0.696-0.958)	0.013	1.000
*RP11-452H21.4*_B_Mem	Carotid plaque build (GCST90503076)	0.844 (0.717-0.993)	0.041	1.000
*TBC1D4*_CD4_ET	Carotid plaque (GCST007435)	0.864 (0.753-0.992)	0.038	1.000
*TBC1D4*_CD4_ET	Carotid plaque build (GCST90503076)	0.845 (0.729-0.979)	0.025	1.000
*TBC1D4*_CD8_NC	Carotid plaque (GCST007435)	0.794 (0.669-0.943)	0.008	0.996
*TBC1D4*_CD8_NC	Carotid plaque build (GCST90503076)	0.822 (0.688-0.983)	0.031	0.998
*TTC39B*_B_IN	Carotid plaque (GCST007435)	0.849 (0.746-0.967)	0.013	0.995
*TTC39B*_B_IN	Carotid plaque build (GCST90503076)	0.823 (0.712-0.950)	0.008	1.000
*VCL*_CD8_NC	Carotid plaque (GCST007435)	0.894 (0.817-0.978)	0.014	0.999
*VCL*_CD8_NC	Carotid plaque build (GCST90503076)	0.911 (0.832-0.998)	0.045	1.000

This table presents the Mendelian randomization (MR) association results between immune cell-specific hub gene expression (Exposure) and carotid plaque or its formation (Outcome). All MR analyses were performed using the Wald ratio method with a single instrumental SNP per exposure-outcome pair. PPH3, posterior probability of hypothesis 3; PPH4, posterior probability of hypothesis 4.

### 3.3 Results of single-cell RNA sequencing analysis

**Figure 2:**
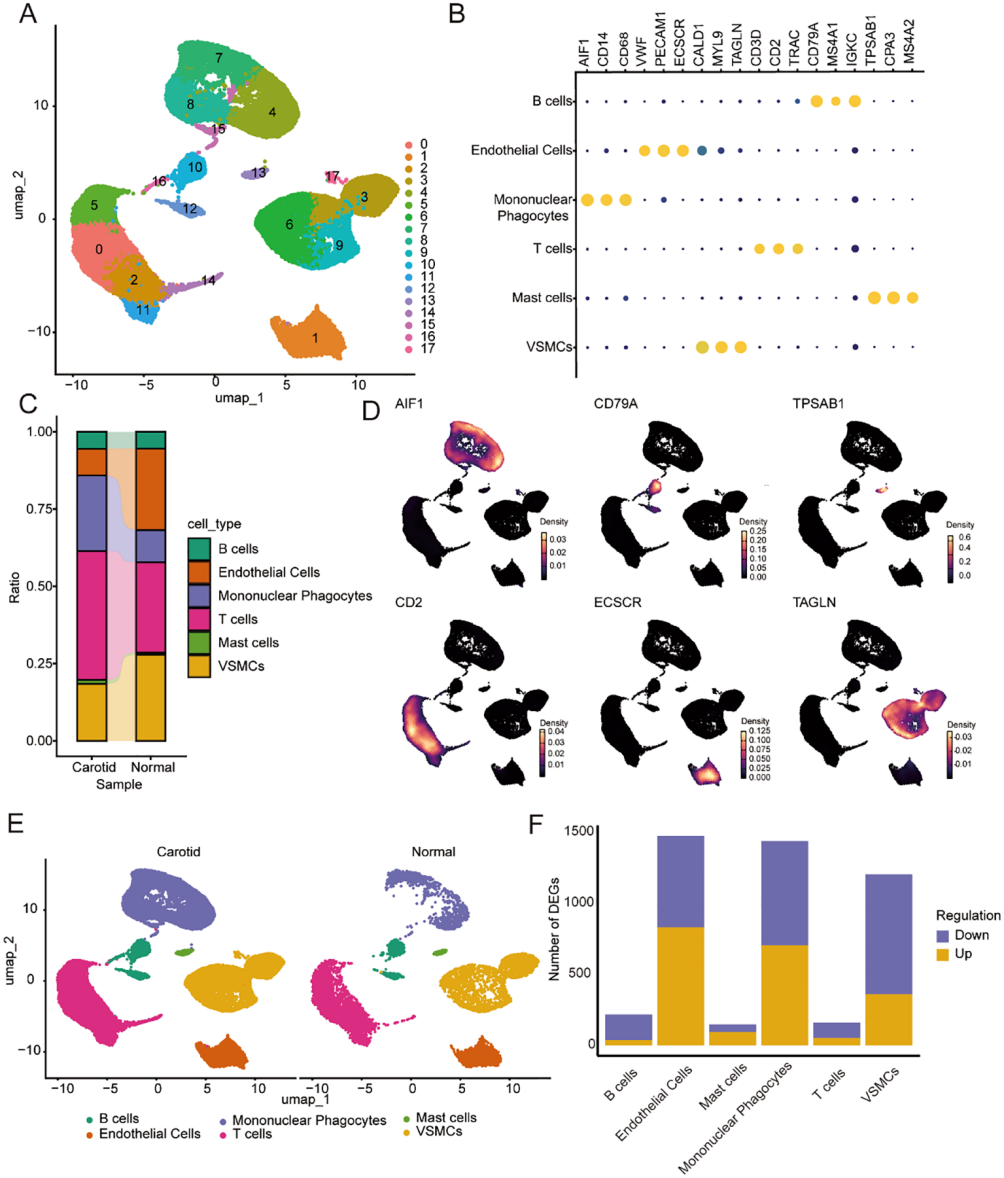
Single-cell RNA sequencing analysis reveals the cellular landscape and *LIPA* expression in carotid plaque. **(A)** Uniform Manifold Approximation and Projection (UMAP) plot showing the unsupervised clustering of 18 distinct cell populations from carotid plaque tissues. **(B)** Dot plot displaying the expression levels of canonical marker genes across major cell types; the color gradient indicates average expression, and the dot size represents the percentage of expressing cells. **(C)** Stacked bar plot quantifying the relative proportion of major cell types (B cells, T cells, mononuclear phagocytes, etc.) in carotid plaque versus normal arterial tissues. **(D)** UMAP feature plots illustrating the spatial expression patterns of key marker genes (AIF1, CD79A, TPSAB1, CD2, ECSCR, TAGLN) defining specific cell clusters. **(E)** UMAP plots showing the distribution of major cell types in carotid plaque and normal samples. **(F)** Bar plot showing the number of differentially expressed genes (DEGs) (upregulated in orange, downregulated in blue) in AC compared to PA across different cell types.

To characterize cell type and state differences between AC and PA samples, 18 cell clusters were first annotated (**Figure 2A**). Marker genes of each cluster were screened and matched to the CellMarker database (xteam.xbio.top/CellMarker/) and literature-reported cell-type markers, ultimately identifying 6 major cell subsets: B cells (*IGKC*, *CD79A*, *MS4A1*), endothelial cells (*ECSCR*, *PECAM1*, *VWF*), mononuclear phagocytes (*AIF1*, *CD14*, *CD68*), T cells (*CD2*, *TRAC*, *CD3D*), mast cells (*TPSAB1*, *CPA3*, *MS4A2*), and vascular smooth muscle cells (VSMCs) (*CALD1*, *MYL9*, *TAGLN*) (**Figures 2B, D, E**). AC and PA samples differed in cell composition (**Figure 2C**): mononuclear phagocyte and T cell proportions were elevated in AC, while endothelial cell and VSMC proportions were reduced. Endothelial cells and mononuclear phagocytes had the most DEGs (>1,400 each), indicating extensive transcriptional alterations in these cell types in AC (**Figure 2F**).

**Figure 3:**
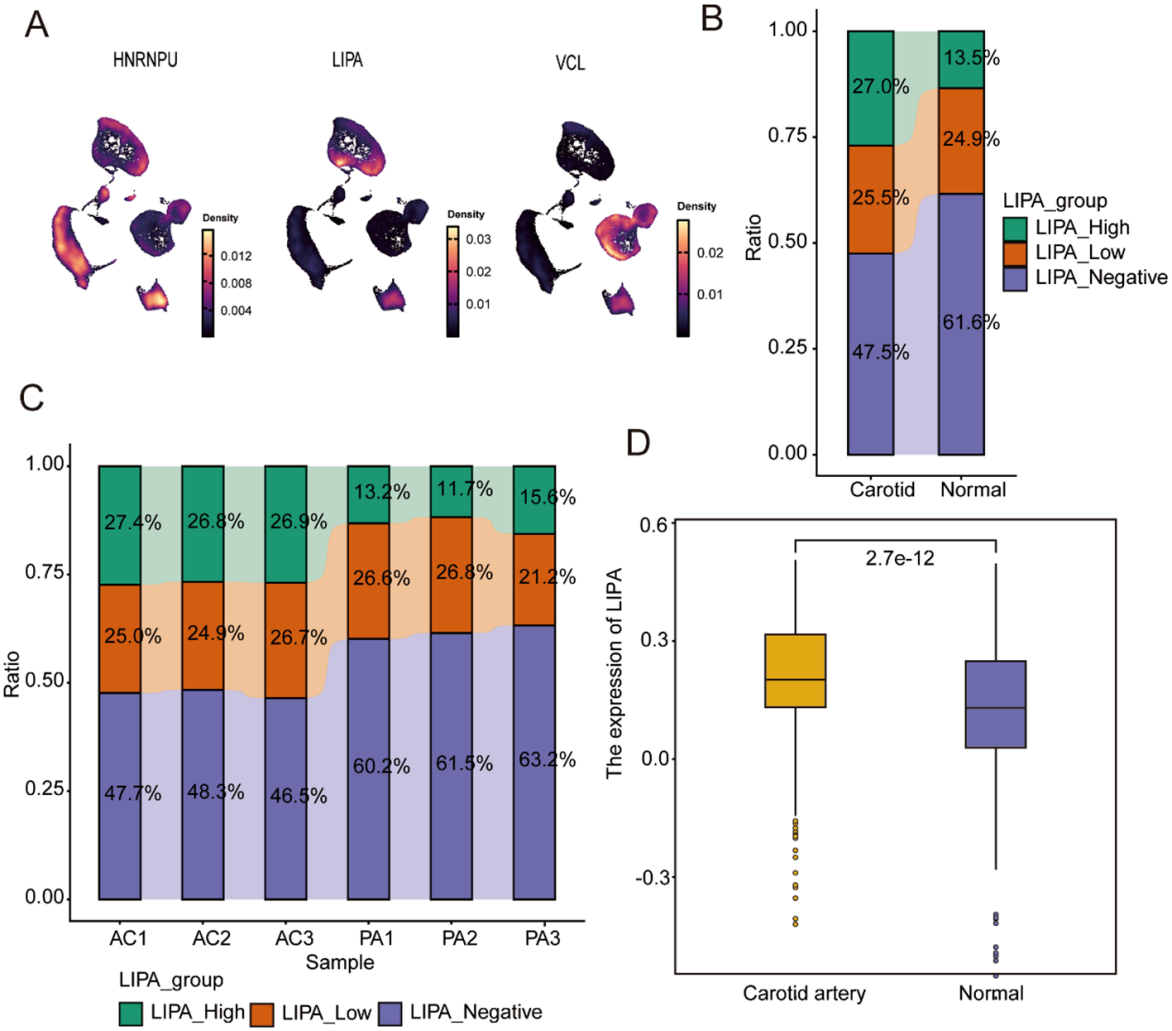
Single-cell RNA sequencing analysis characterizes the expression patterns of MR-prioritized candidate genes and *LIPA* stratification. **(A)** UMAP feature plots showing the spatial expression distribution of three core causal genes (*HNRNPU*, *LIPA*, *VCL*) identified by MR analysis in carotid plaque tissues. **(B)** Stacked bar plot comparing the relative proportions of *LIPA* expression subgroups (High, Low, Negative) between carotid plaque and normal arterial tissues. **(C)** Stacked bar plot quantifying the *LIPA* expression stratification across individual carotid plaque (AC1-AC3) and normal (PA1-PA3) samples. **(D)** Box plot showing the expression levels of *LIPA* in atherosclerotic calcified carotid plaque (AC) and paired normal arterial (PA) tissues; the difference was statistically significant (*P* = 2.7×10^-^¹², Wilcoxon rank-sum test).

Integrated MR and scRNA-seq analysis identified three candidate genes: *LIPA* was upregulated, and *HNRNPU* and *VCL* downregulated, in AC vs. PA (consistent with CP causal associations; **Supplementary Figure 6**). *HNRNPU* was highly expressed in mononuclear phagocytes, endothelial cells, T cells, and mast cells; *LIPA* in mononuclear phagocytes and endothelial cells; and *VCL* in VSMCs and endothelial cells (**Figure 3A**). *LIPA* was highly expressed in AC mononuclear phagocytes (**Supplementary Figure 7**), matching its MR-associated Mono_C cell type and its prioritization in the Mono_C genetic context and enrichment in plaque mononuclear phagocytes. Wilcoxon rank-sum tests confirmed significantly higher *LIPA* expression in AC vs. PA (*P* < 0.01, **Figure 3D**), leading to its selection as the lead functional candidate enriched in plaque mononuclear phagocytes.

*LIPA* expression in mononuclear phagocytes was stratified into Negative, Low, and High groups. AC had a higher proportion of High *LIPA*-expressing cells (27.0%) than PA (13.5%), with a similar trend for Low expression (**Figures 3B, C**). This indicated increased *LIPA*-expressing mononuclear phagocytes in pathological CP, supporting the co-occurrence of elevated *LIPA* expression and mononuclear phagocyte expansion in plaque tissues.

**Figure 4:**
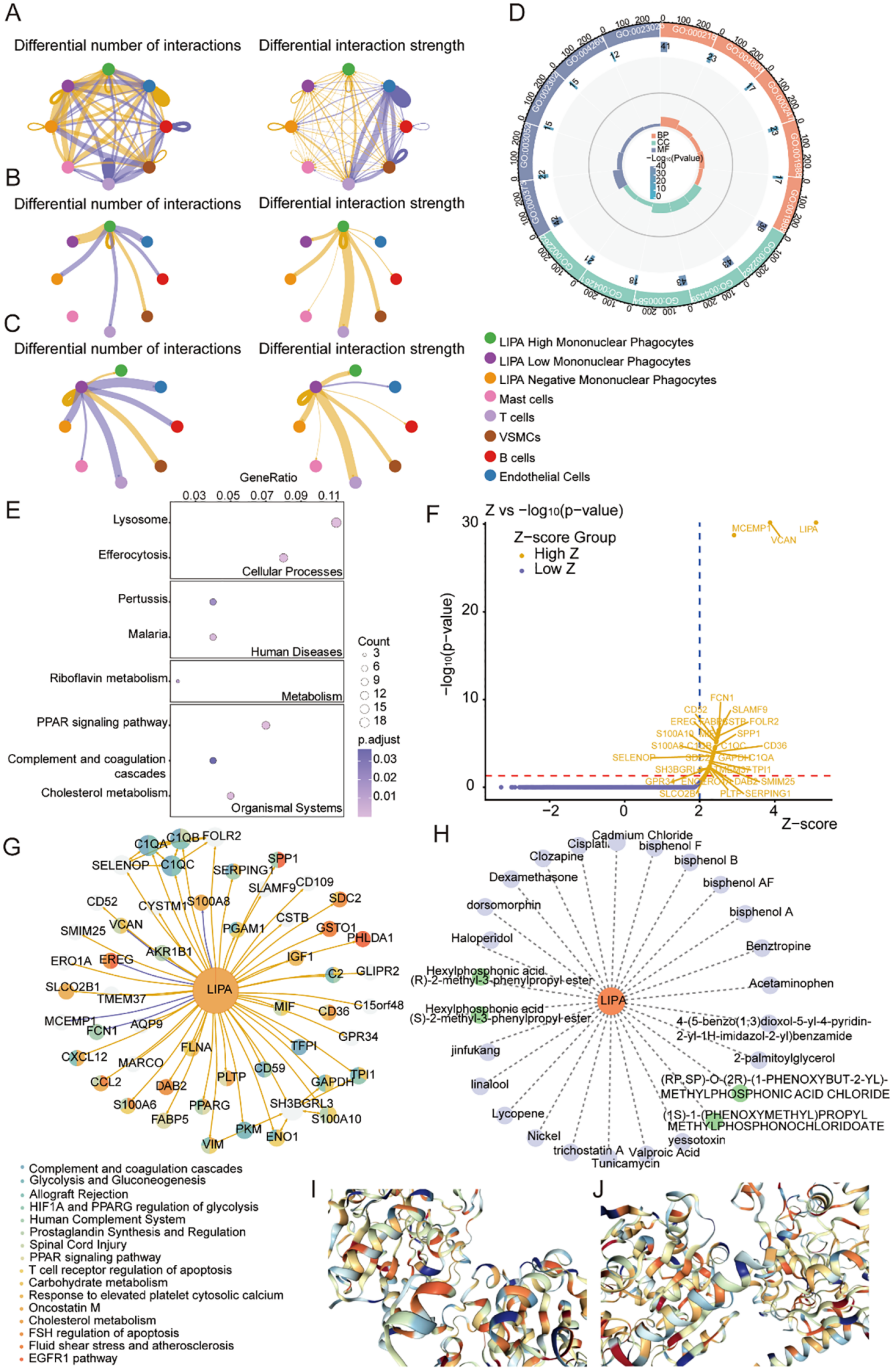
*LIPA*-mediated intercellular communication, functional enrichment, in silico knockout and targeted drug prediction in carotid plaque. **(A)** Circular plot showing differential intercellular communication between atherosclerotic calcified carotid plaque (AC) and paired normal proximal tissue (PA) groups; left panel for differential interaction numbers and right panel for differential interaction intensity (yellow, increased in AC; purple, decreased in AC). **(B)** Circular plot of differential intercellular communication profiles of *LIPA*-high mononuclear phagocytes in AC vs. PA groups. **(C)** Circular plot of differential intercellular communication profiles of *LIPA*-low mononuclear phagocytes in AC vs. PA groups. **(D)** Dot plot of Gene Ontology (GO) functional enrichment analysis for differentially expressed genes (DEGs) in *LIPA*-expressing mononuclear phagocytes. **(E)** Dot plot of Kyoto Encyclopedia of Genes and Genomes (KEGG) pathway enrichment analysis for DEGs in *LIPA*-expressing mononuclear phagocytes. **(F)** Scatter plot showing the degree of gene expression perturbation in mononuclear phagocytes after virtual *LIPA* knockout via scTenifoldKnk. **(G)** Functional enrichment analysis of genes with significantly altered expression post *LIPA* in silico knockout. **(H)** *LIPA*-drug interaction network; purple nodes represent drugs from the Comparative Toxicogenomics Database (CTD), green nodes from DrugBank, and the orange node represents LIPA protein. **(I)** Molecular docking model of LIPA protein with dexamethasone. **(J)** Molecular docking model of LIPA protein with dorsomorphin.

Cell communication analysis (**Figures 4A–C**) revealed that, in AC vs. PA groups, *LIPA*-expressing mononuclear phagocytes exhibited higher interaction intensity but fewer interactions with T cells, with an SPP1-CD44 ligand-receptor interaction predicted in AC but not PA in this dataset (**Supplementary Figures 8, S9**). This suggests *LIPA*-expressing mononuclear phagocytes and T cells may contribute to CP formation and progression by enhancing specific interaction intensity and regulating the *SPP1*-*CD44* pathway. Additionally, the AC group specifically activated the DHEAS, PLAU, IGFBP, ApoE, PTN, and SPP1 signaling pathways (**Supplementary Figure 10**), which were inactive or weakly activated in PA and may be associated with CP-related immune remodeling. In contrast, the PA group exhibited activation of homeostasis-maintaining pathways (SELE, COMPLEMENT; **Supplementary Figure 10**), reflecting the homeostatic regulatory features of the normal tissue microenvironment.

Differential expression analysis of mononuclear phagocytes stratified by *LIPA* expression identified 290 DEGs (126 highly expressed in the *LIPA*-negative subset, 164 in the *LIPA*-positive subset). Functional enrichment analysis of these DEGs showed GO enrichment primarily in inflammatory response regulation (GO:0050727), secretory granule lumen (GO:0034774), and lipoprotein particle binding (GO:0071813) (**Figure 4D**). KEGG pathway enrichment revealed major involvement in Lysosome, Cholesterol metabolism, and the PPAR signaling pathway (**Figure 4E**).

Immune microenvironment characteristics were compared between atherosclerotic and normal carotid tissues. Immune infiltration analysis revealed significantly elevated proportions of B lineage, CD8 T cells, cytotoxic lymphocytes, monocytic lineage, myeloid dendritic cells, and T cells in atherosclerotic samples (*P* < 0.05, **Supplementary Figure 11A**). *LIPA* expression was positively correlated with monocytic lineage proportion (*P* < 0.01, **Supplementary Figure 11B**), suggesting its involvement in CP immune-inflammatory processes and that its expression reflects the monocytic lineage infiltration degree from bulk-tissue deconvolution.

Immune function analysis showed that Type II IFN Response activity was significantly lower in the atherosclerotic group, while aDCs, APC co-inhibition, APC co-stimulation, and B cell pathway activities were higher (*P* < 0.05, **Supplementary Figure 11C**). *LIPA* expression was positively correlated with parainflammation, macrophages, APC co-stimulation, and checkpoint (*P* < 0.05, **Supplementary Figure 11D**), suggesting that *LIPA* expression is associated with immune functional modules involved in plaque microenvironment remodeling. Collectively, single-cell and bulk-tissue immune analyses provide convergent associative support for the MR-prioritized role of *LIPA*.

To further explore *LIPA*’s regulatory role in mononuclear phagocytes, in silico knockout was performed in this subset via *scTenifoldKnk* using single-cell transcriptome data, identifying 53 genes with significantly altered expression (p.adjust < 0.05, **Supplementary Table 10**; **Figure 4F**). Functional enrichment analysis of these 53 genes revealed enrichment in the Complement and coagulation cascades, Glycolysis and Gluconeogenesis, Allograft Rejection, HIF1A/PPARG-mediated glycolysis regulation, Human Complement System, and Prostaglandin Synthesis and Regulation pathways (**Figure 4G**). Most of these pathways are involved in immune-inflammatory activation, cellular metabolic remodeling, and lipid homeostasis regulation (27–29), suggesting *LIPA* may modulate the functional phenotype of mononuclear phagocytes via these core pathways to participate in CP pathogenesis.

### 3.4 Drug prediction, molecular docking, and PheWAS results

To evaluate the clinical application potential of core gene *LIPA* in CP initiation and progression, we screened *LIPA*-targeting small molecules via DrugBank and CTD, yielding 4 and 22 potential drugs, respectively (**Figure 4H**; **Supplementary Table 11**). Molecular docking showed *LIPA* had high binding affinity with 16 candidates (**Supplementary Figure 12**; **Supplementary Table 12**); dorsomorphin (Vina score = -8) and dexamethasone (Vina score = -7.5) may exert CP therapeutic effects by targeting *LIPA* (**Figures 4I, J**). While both dorsomorphin (Vina score = –8) and dexamethasone (Vina score = –7.5) showed favorable binding scores, dorsomorphin was prioritized for *in vitro* validation only as a pharmacological tool to explore LIPA−associated biology, based on its reported activity in lipid metabolism and complement regulation pathways, as well as its commercial availability and well−characterized cellular effects relevant to foam cell biology. Additionally, PheWAS analysis did not reveal strong pleiotropic associations in the tested phenotypes (**Supplementary Figures 13, S14**, **Supplementary Tables 13, S14**).

### 3.5 *In vitro* experimental validation of *LIPA* function and drug targeting in macrophages

To validate the functional role of *LIPA* suggested by our multi-omics analyses and explore the pharmacological perturbation of the lead candidate, we performed a series of *in vitro* experiments using THP-1-derived macrophages and Jurkat T cells.

**Figure 5:**
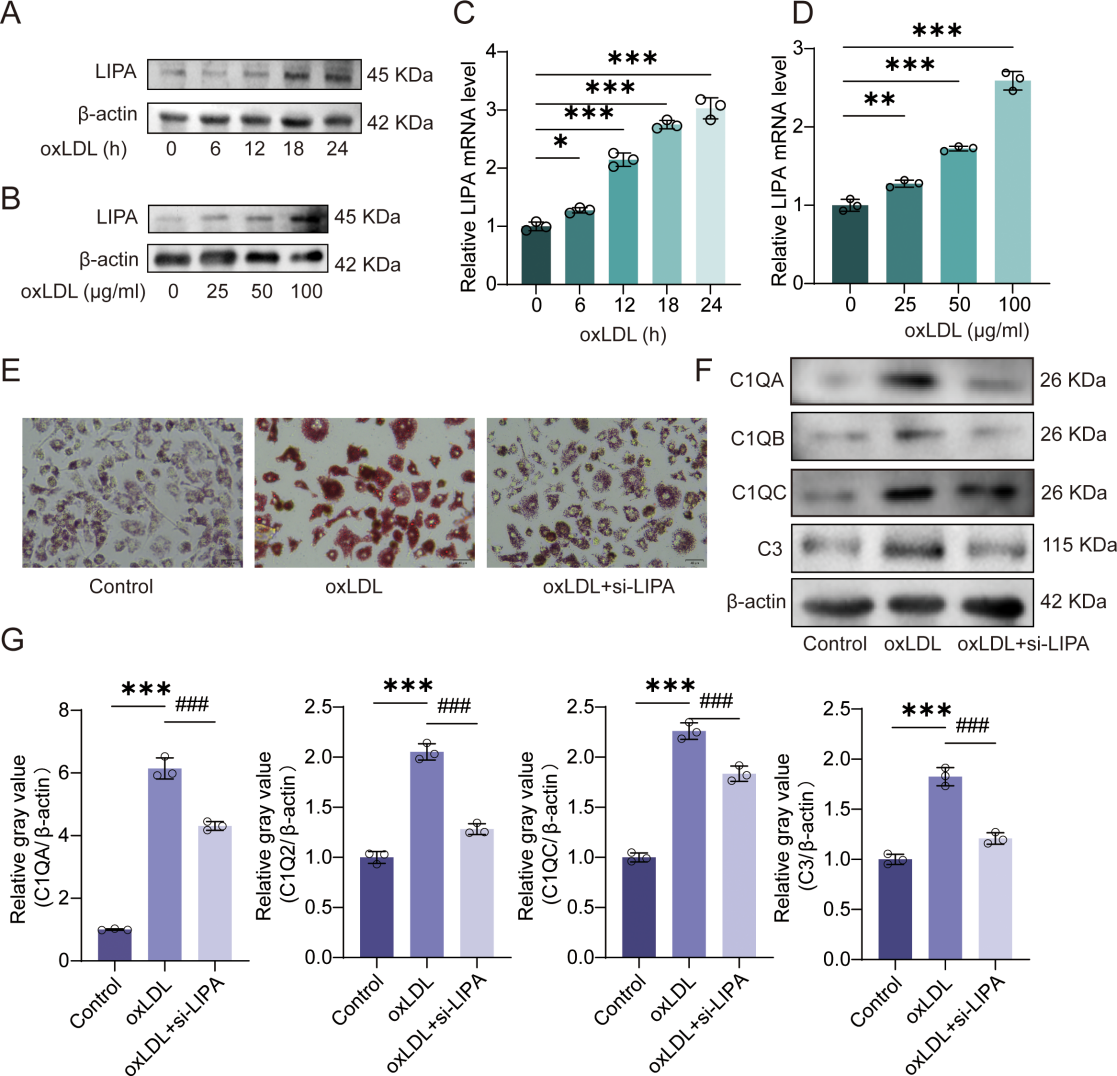
OxLDL upregulates *LIPA* expression in a time- and dose-dependent manner, and *LIPA* knockdown attenuates oxLDL-induced complement activation in THP-1-derived macrophages. **(A, B)** Western blot analysis of LIPA protein expression in THP-1-derived macrophages treated with 100 μg/mL oxLDL for different time points (0-24 h) **(A)** or different concentrations of oxLDL (0-100 μg/mL) for 24 h **(B)**. **(C, D)** qRT-PCR analysis of *LIPA* mRNA expression in THP-1-derived macrophages treated with oxLDL in a time-dependent **(C)** and dose-dependent **(D)** manner. Data are mean ± SD, *P < 0.05, **P < 0.01, ***P < 0.001. **(E)** Representative Oil Red O staining of lipid accumulation in macrophages with or without *LIPA* knockdown under oxLDL stimulation. Scale bar: 40 μm. **(F, G)** Western blot and quantitative analysis of complement proteins (C1QA, C1QB, C1QC, C3) in macrophages. Data are mean ± SD, ***P < 0.001 vs. control; *###P < 0.001* vs. oxLDL group.

We examined how oxidized low-density lipoprotein (oxLDL), a major factor in atherosclerosis, influences *LIPA* expression. OxLDL treatment of THP-1-derived macrophages significantly increased LIPA protein (**Figures 5A, B**) and mRNA levels (**Figures 5C, D**). This finding corroborates the pathological upregulation of *LIPA* observed in carotid plaque mononuclear phagocytes.

We next explored the functional consequences of *LIPA* dysregulation. Knockdown of *LIPA* (si-*LIPA*) in oxLDL-stimulated (100 μg/ml, 24h) macrophages markedly reduced intracellular lipid accumulation, as evidenced by Oil Red O staining (**Figure 5E**). Furthermore, in silico knockout predictions were experimentally validated by showing that *LIPA* deficiency significantly attenuated the oxLDL-induced upregulation of complement system components C1QA, C1QB, C1QC, and C3 (**Figure 5F**). This suggests a candidate pathway link between *LIPA*-mediated lipid metabolism and innate immune activation within the plaque environment.

**Figure 6:**
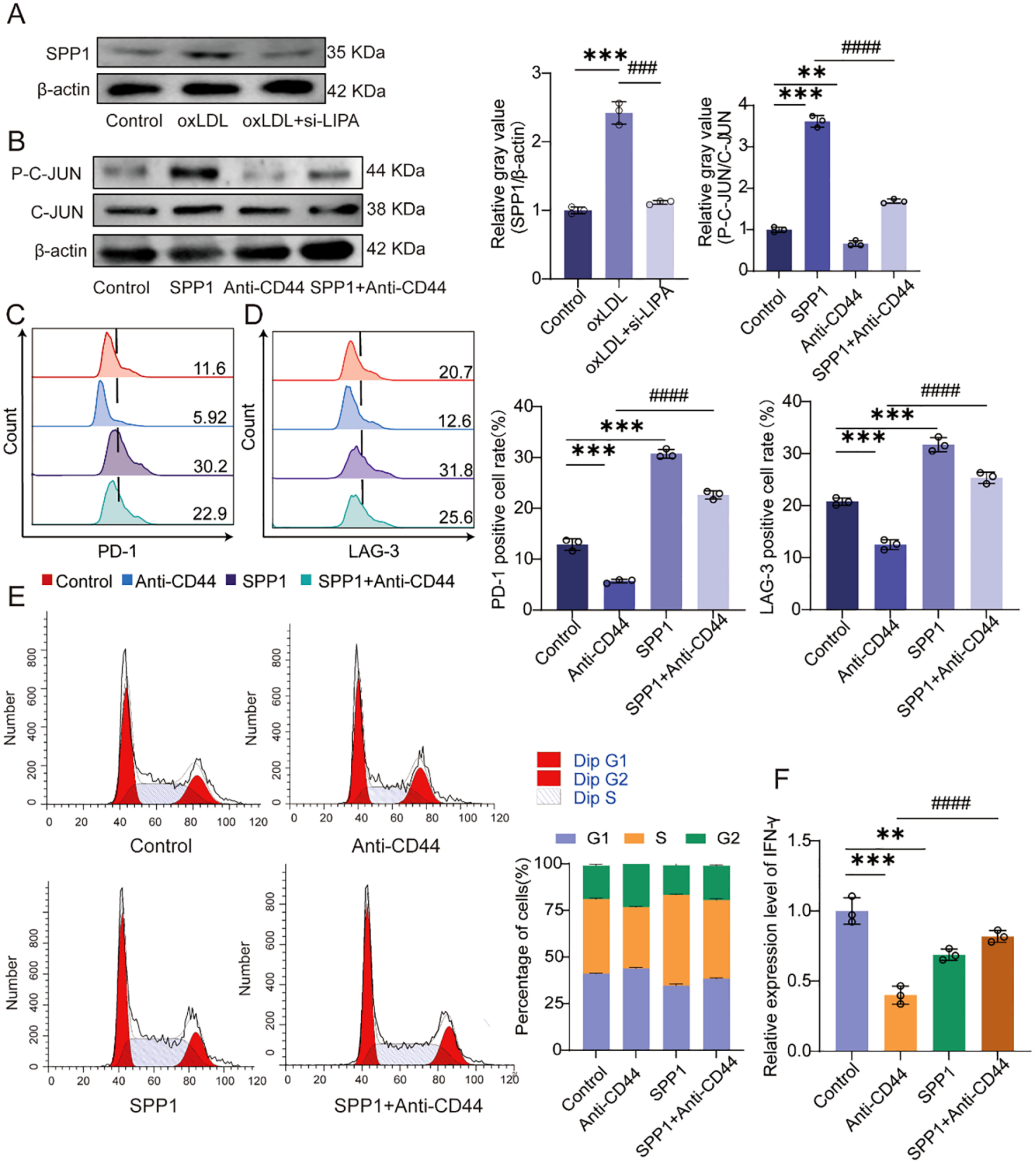
SPP1/CD44-associated signaling influences macrophage c-JUN phosphorylation and T-cell functional readouts *in vitro*. **(A)** Western blot analysis and quantitative densitometry of SPP1 protein expression in THP-1-derived macrophages treated with control, oxLDL, or oxLDL plus si-*LIPA*. β-actin served as the loading control. **(B)** Western blot detection and quantification of phosphorylated c-JUN (p-c-JUN) and total c-JUN in THP-1-derived macrophages treated with control, SPP1, Anti-CD44, or SPP1 plus Anti-CD44. β-actin was used as an internal reference. **(C, D)** Flow cytometric analysis and quantification of PD-1 (programmed cell death protein 1) **(C)** and LAG-3 (lymphocyte activation gene 3) **(D)** expression in Jurkat T cells collected from the upper chamber after THP-1 macrophage–Jurkat T cell Transwell co-culture under the indicated treatments. **(E)** Representative flow cytometry plots and statistical analysis of cell cycle distribution (G1, S, and G2 phases) in Jurkat T cells collected after Transwell co-culture. **(F)** ELISA-based measurement of IFN-γ secretion in co-culture supernatants under the indicated treatments. Data are presented as mean ± SD, ***P < 0.001 vs. Control; *####P < 0.0001* vs. SPP1 group.

We next examined whether the predicted SPP1-CD44 axis was supported by *in vitro* functional readouts. Western blot analysis confirmed that oxLDL significantly induced SPP1 expression in macrophages, an effect that was largely abolished by *LIPA* knockdown (**Figure 6A**). We then examined the downstream signaling and found that recombinant SPP1 protein robustly enhanced the phosphorylation of c-JUN, whereas neutralization of CD44 with a specific antibody markedly abrogated this induction, indicating that CD44 blockade attenuated SPP1-induced c-JUN phosphorylation under these experimental conditions (**Figure 6B**). Functionally, flow cytometric analyses demonstrated that SPP1 treatment increased the proportions of PD−1^+^ and LAG−3^+^ T cells, consistent with an exhaustion-associated phenotype; this effect was significantly reversed by CD44 blockade (**Figures 6C, D**). Consistent with the immunomodulatory phenotypes, cell cycle profiling revealed that SPP1 reduced the G1 phase population while increasing the S phase fraction, thereby promoting cell cycle progression; these changes were significantly suppressed by CD44 inhibition (**Figure 6E**). Moreover, in the Transwell co-culture system, SPP1 significantly diminished IFN-γ secretion in the co-culture supernatants, whereas CD44 blockade partially restored IFN-γ levels, supporting an immunomodulatory role of the SPP1-CD44 axis (**Figure 6F**). Together, these data support a candidate pathway in which LIPA-associated SPP1 upregulation in macrophages may influence CD44-dependent T-cell functional readouts in a macrophage–T cell Transwell co-culture setting.

**Figure 7:**
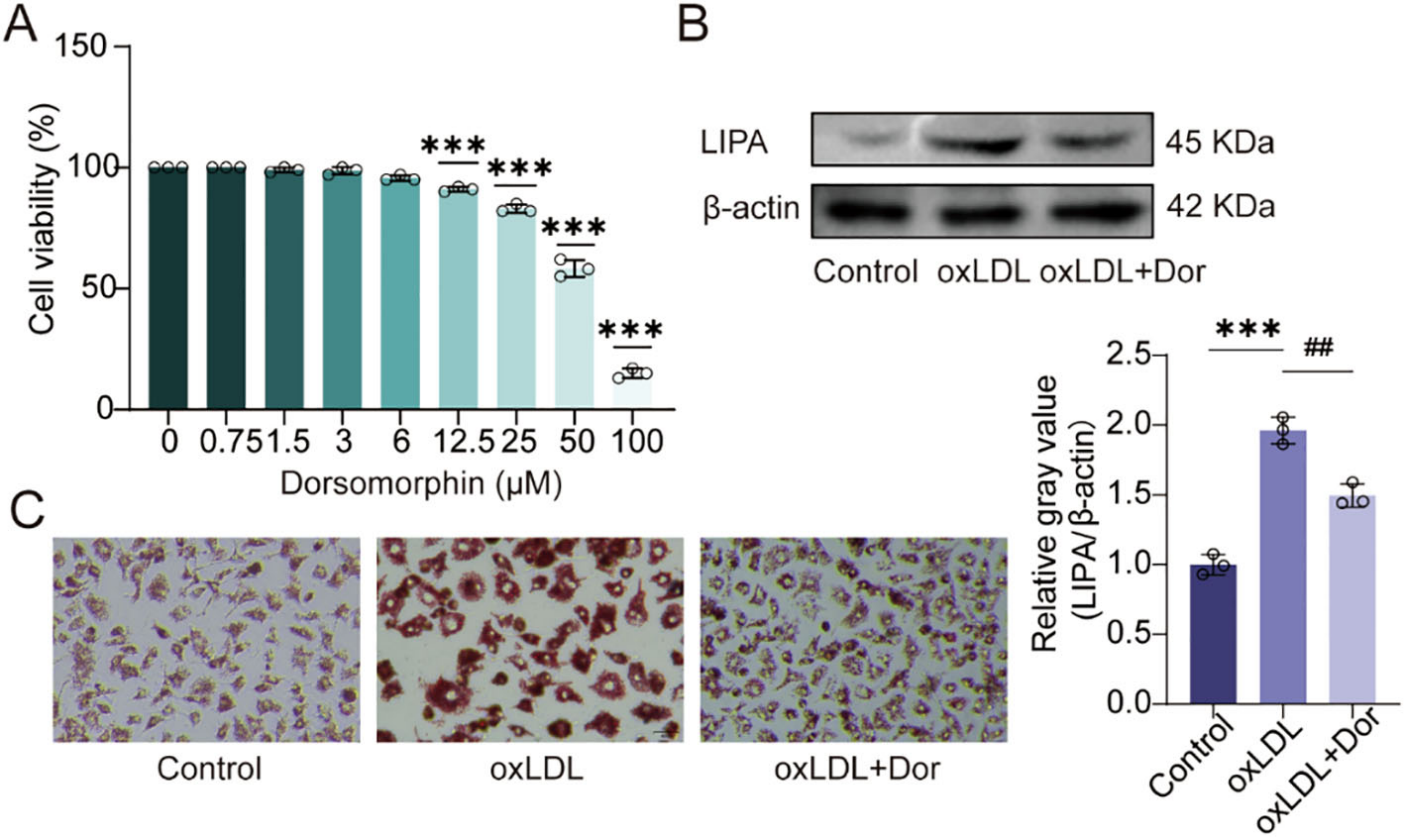
Dorsomorphin attenuates oxLDL-Induced *LIPA* Upregulation in THP-1-Derived Macrophages. **(A)** The viability of THP-1-derived macrophages treated with increasing concentrations of Dorsomorphin (0, 0.75, 1.5, 3, 6, 12.5, 25, 50, 100 μM) for 24 h. Data are presented as mean ± SD (n = 3), ***P < 0.001 vs. 0 μM group (one-way ANOVA followed by Tukey’s *post hoc* test). **(B)** Western blot analysis (top) and quantitative gray value analysis (bottom) of LIPA protein expression in THP-1-derived macrophages divided into three groups: Control, oxLDL, and oxLDL + Dorsomorphin. β-actin was used as the loading control. Data are presented as mean ± SD, ***P < 0.001 vs. Control group; *##P < 0.01* vs. oxLDL group (unpaired t-test or one-way ANOVA). **(C)** Representative Oil Red O staining images of lipid accumulation in THP-1-derived macrophages from the Control, oxLDL, and oxLDL + Dorsomorphin groups. Scale bar: 40 μm.

Subsequently, we tested dorsomorphin as a pharmacological perturbation tool, a compound identified through molecular docking. Treatment of oxLDL-stimulated macrophages with a non-cytotoxic concentration of dorsomorphin (**Figure 7A**) significantly reduced *LIPA* protein expression (**Figure 7B**) and correspondingly decreased intracellular lipid accumulation (**Figure 7C**). This provides preliminary *in vitro* evidence supporting that reduction of oxLDL-induced *LIPA* upregulation was accompanied by attenuated lipid accumulation; target specificity requires further validation.

## 4 Discussion

CP remains the leading cause of ischemic stroke, yet the cell type-specific genetic regulatory networks underlying its pathogenesis have not been fully elucidated (1, 30). Atherosclerosis exhibits pronounced vascular bed specificity, but the majority of genetic studies to date have focused on coronary artery disease. Furthermore, dysfunction of mononuclear phagocytes—the core effector cells in the pathological progression of atherosclerotic plaques—is a key driver of inflammatory amplification and plaque destabilization (8). By integrating sc-eQTL-driven MR and single-cell transcriptomics, our study provides the evidence prioritizing *LIPA* as a mononuclear phagocyte-enriched candidate gene associated with CP risk.

Our findings align with and extend the work of Li et al., demonstrating that *LIPA* is significantly upregulated in plaque-associated phagocytes and regulates lysosomal and cholesterol metabolism (31), consistent with its established role encoding lysosomal acid lipase (32, 33). Critically, we revealed that MR signal was prioritized in the Mono_C context, while scRNA-seq showed enrichment of *LIPA* in plaque mononuclear phagocytes. The increased proportion of *LIPA*-expressing mononuclear phagocytes in CP tissue compared with paired adjacent normal arterial tissues indicates that *LIPA* may serve as a candidate marker of a plaque-associated phagocyte state potentially linked to plaque instability. Meanwhile, the altered cellular composition we identified in CP tissue—including elevated proportions of mononuclear phagocytes and T cells, and reduced proportions of VSMCs and endothelial cells—is fully consistent with previous single-cell transcriptomic studies of CP (34).

Beyond its canonical role in lipid metabolism, our study uncovered a potential association between *LIPA* perturbation and complement/coagulation-related transcriptional programs. Accumulating evidence has established that dysregulation of the complement cascade is closely linked to the maintenance of low-grade chronic inflammation and the recurrent flares observed in a spectrum of inflammatory pathologies (35). Through in silico knockout simulation, we found that *LIPA* deficiency significantly modulated these pathways in mononuclear phagocytes. While traditional views have focused on systemic complement activation, recent studies have revealed that intracellular complement signaling in macrophages is the cornerstone of sustained cellular activation in atherosclerosis (36). Macrophages, one of the most important subsets of mononuclear phagocytes, are central mediators of the inflammatory response in atherosclerosis. They not only promote atherogenesis by regulating M1/M2 macrophage polarization, but are also functionally modulated by the complement system (9). Wang et al. recently discovered that elevated PLA2G2A levels in vascular fibroblasts accelerate plaque progression by triggering complement and coagulation pathways in macrophages (35). Kiss et al. found that high intracellular C3 in macrophages slows atherosclerosis by hindering macrophage efferocytosis (37). Thus, understanding the complex roles of the complement system in atherosclerosis is crucial. Our data suggest that *LIPA* may act as a mechanistic link between metabolic dysfunction and excessive innate immune activation, providing a potential explanation for the amplified local inflammatory response observed in CP. This finding expands the functional scope of *LIPA* from a purely metabolic enzyme to a candidate modulator of plaque immune-metabolic remodeling.

Aberrant intercellular communication between immune cells is a core mechanism driving the remodeling of the CP immune microenvironment, and represents a key research frontier in the field of atherosclerosis (8). In the microenvironment of progressive plaques, mononuclear phagocytes act as initiators of the inflammatory response, engaging in bidirectional crosstalk with T cells via cytokines, chemokines, and membrane-bound ligand-receptor signaling. This aberrant intercellular communication not only directly amplifies the local inflammatory cascade but also induces pro-inflammatory polarization of T cells, promotes macrophage foam cell formation, and accelerates extracellular matrix degradation, collectively driving plaque destabilization (38). However, current research on immune cell crosstalk in CP tissue remains limited. Previous studies have mostly focused on generalized inflammatory pathways and have yet to delineate the specific communication axis mediated by pathogenic phagocyte subpopulations in CP. This study highlights the discovery of a crosstalk network between *LIPA*-expressing mononuclear phagocytes and T cells in CP tissue, with the SPP1-CD44 ligand-receptor axis identified as a key mediator. We found that this signaling axis was markedly enriched in CP tissue, whereas no such activation was observed in paired adjacent normal vascular tissues. Earlier research has shown that SPP1 plays a crucial role in attracting macrophages and causing plaque instability (39, 40), while CD44 is a major adhesion molecule and signaling receptor on the surface of T cells that regulates T cell migration, proliferation, and pro-inflammatory differentiation in response to SPP1 stimulation (41). Our findings extend this model, demonstrating that this signaling axis is predominantly active in CP and may contribute to pro-inflammatory signal transduction from *LIPA*-expressing mononuclear phagocytes to T cells. This is the first study to link the SPP1-CD44 signaling axis to the *LIPA*-expressing pathogenic phagocyte subpopulation in CP. This interaction may promote T cell recruitment and polarization, potentially forming a positive feedback loop that contributes to matrix degradation and increased plaque vulnerability. Recently, Nie et al. reported that macrophage-secreted SPP1 promotes CP formation by inducing foam cell formation and the release of pro-inflammatory cytokines (42). A prospective cohort study by Wang indicated that elevated circulating SPP1 is a predictive biomarker of immune-inflammatory plaque instability (43). In addition, SPP1-positive macrophages have been widely identified in various human malignancies, and targeting this macrophage subpopulation has emerged as a promising anti-cancer therapeutic avenue (44). Our *in vitro* experiments further substantiate these findings.

Aligned with multi-omics predictions, we verified that oxLDL stimulation directly increases *LIPA* expression in macrophages, depending on time and dose, similar to the high *LIPA* levels seen in carotid plaque mononuclear phagocytes. Knockdown of *LIPA* not only attenuated oxLDL-induced lipid accumulation but also suppressed the expression of complement components C1QA, C1QB, C1QC, and C3, providing experimental validation for the complement and coagulation cascade enrichment predicted by in silico knockout. Moreover, *LIPA* deficiency markedly reduced SPP1 expression in oxLDL-stimulated macrophages, and our co-culture experiments demonstrated that SPP1 promotes T cell exhaustion and proliferation via CD44-mediated activation of the c-JUN pathway. These functional assays provide preliminary support for a *LIPA*-SPP1/CD44-related pathway between *LIPA*-mediated lipid dysregulation and the remodeling of the plaque immune microenvironment. Dorsomorphin successfully inhibited oxLDL-induced LIPA increase and foam cell formation, providing initial evidence for targeting *LIPA* pharmacologically in atherosclerosis. Notably, in addition to mononuclear phagocytes, *LIPA* was also expressed in endothelial cells—the cell type with the most extensive transcriptional differences between AC and PA. This suggests a potential role for endothelial *LIPA* in plaque pathophysiology, possibly through lipid handling or inflammatory modulation. However, its functional significance remains unclear, and future studies using endothelial−specific models are needed to dissect its relative contribution.

The study also yielded several exploratory secondary findings that warrant further independent investigation. In addition to *LIPA*, we also identified *HNRNPU* and *VCL* as novel high-confidence candidates for CP. The significant downregulation of *HNRNPU*, an RNA-binding protein involved in post-transcriptional RNA regulation, suggests that epigenetic and transcriptional dysregulation may contribute to CP progression (45, 46). Previous studies have similarly found decreased VCL expression in atherosclerotic plaque tissue (47). Furthermore, the identification of B-cell-specific candidate genes may motivate exploratory studies of B-cell-related immune regulation. These include *TTC39B* (48), which has been previously validated to regulate lipid metabolism and atherogenesis, as well as two novel memory B cell-associated lncRNA transcripts, *RP1-313I6.12* and *RP11-452H21.4*.

In the exploratory translational pharmacological arm of our study, our molecular docking analysis identified 16 small-molecule compounds with potential binding affinity to *LIPA*. Among them, dexamethasone exhibited high binding affinity to *LIPA*, suggesting that its well-documented anti-atherosclerotic and anti-inflammatory effects may be partially mediated via the modulation of *LIPA* (49). Conversely, the binding of antipsychotic drugs such as haloperidol and clozapine to *LIPA* provides a potential molecular mechanism underlying the metabolic side effects and increased CP risk associated with these medications (50). These findings underscore *LIPA*’s potential as a dual-purpose pharmacological target for drug repurposing and safety screening. However, the predicted interactions between these compounds and *LIPA* need further validation through detailed *in vitro* and *in vivo* experiments to confirm target specificity and therapeutic efficacy in CP. Building on these preliminary in silico findings, *LIPA*-targeted modulation represents an exploratory therapeutic concept, with potential relevance for addressing residual inflammation in inflammatory carotid plaques, pending further preclinical validation.

Several limitations of the present study merit discussion. First, our genetic and transcriptomic evidence has inherent dataset-related constraints. The single-cell and immune microenvironment analyses were cross-sectional and therefore could not capture the spatiotemporal dynamics of LIPA expression during CP progression. In addition, the sc-eQTL and GWAS datasets were mainly derived from European populations, and the sc-eQTL data originated from peripheral blood immune cells rather than plaque-resident mononuclear phagocytes, which may limit the generalizability and tissue-specific interpretation of our findings. Second, although the SPP1-CD44 axis was supported by single-cell predictions and a THP-1/Jurkat Transwell co-culture system, this study did not systematically examine antigen presentation or broader adaptive immune responses. Moreover, the functional validation was mainly based on THP-1-derived macrophages and Jurkat T cells, and further validation using primary human monocyte-derived macrophages, primary T cells, direct-contact blocking/rescue experiments, and *in situ* plaque studies is needed. Third, all functional experiments were conducted *in vitro*; therefore, *in vivo* validation in atherosclerotic animal models is required to confirm the pathophysiological relevance of LIPA-related mechanisms. Fourth, dorsomorphin has broad activity against multiple targets, including AMPK and BMP pathways, and the observed anti-foam cell effects may partly reflect off-target actions. More specific LIPA-targeting tools or inhibitors, together with direct target-engagement assays such as surface plasmon resonance, are needed to confirm compound binding and target specificity.

## 5 Conclusion

Collectively, by integrating sc-eQTL-driven MR with single-cell transcriptomics and *in vitro* functional assays, we prioritized *LIPA* as a mononuclear phagocyte-enriched candidate gene associated with carotid plaque. *LIPA*-high mononuclear phagocytes were linked to lipid-metabolic remodeling, complement-related responses, and a predicted SPP1-CD44-c-JUN communication axis, which may contribute to plaque immune remodeling. Pharmacological perturbation with dorsomorphin reduced oxLDL-induced *LIPA* upregulation and foam cell formation *in vitro*, providing preliminary support for the translational relevance of *LIPA*-related modulation. These findings nominate *LIPA* for further validation in primary human immune cells, *in vivo* models, and plaque-specific genetic or spatial datasets.

## Data Availability

The original contributions presented in the study are included in the article/**Supplementary Material**. Further inquiries can be directed to the corresponding author.

## Glossary

AC: Atherosclerotic Calcified Carotid Plaque
APC: Antigen-Presenting Cell
B_IN: Naive B Cells
B_Mem: Memory B Cells
CCA: Canonical Correlation Analysis
CD4_NC: Naive CD4^+^
CD8_ET: Effector CD8^+^
CI: Confidence Interval
cis-eQTL: cis-Expression Quantitative Trait Locus
CP: Carotid Plaque
CTD: Comparative Toxicogenomics Database
DEGs: Differentially Expressed Genes
eQTLs: Expression Quantitative Trait Loci
GO: Gene Ontology
GSVA: Gene Set Variation Analysis
GWAS: Genome-Wide Association Study
HVGs: Highly Variable Genes
IFN: Interferon
IVW: Inverse Variance Weighted
IVs: Instrumental Variables
KEGG: Kyoto Encyclopedia of Genes and Genomes
MAF: Minor Allele Frequency
Mono_C: Classical Monocytes
MR: Mendelian Randomization
MR-PRESSO: Mendelian Randomization Pleiotropy RESidual Sum and Outlier
OR: Odds Ratio
oxLDL: Oxidized Low-Density Lipoprotein
PA: Adjacent Normal Proximal Tissue
PCA: Principal Component Analysis
PDB: Protein Data Bank
PBMCs: Peripheral Blood Mononuclear Cells
PheWAS: Phenome-Wide Association Study
PPH3: Posterior Probability of Hypothesis 3
PPH4: Posterior Probability of Hypothesis 4
sc-eQTLs: Single-Cell Expression Quantitative Trait Loci
scRNA-seq: Single-Cell RNA Sequencing
SNP: Single Nucleotide Polymorphism
ssGSEA: Single Sample Gene Set Enrichment Analysis
UMAP: Uniform Manifold Approximation and Projection
VSMCs: Vascular Smooth Muscle Cells
