# Supplementary material for: Integrative genetic and expression profiling prioritizes LIPA in mononuclear phagocytes as a candidate regulator of carotid plaque
Source: Front Immunol. 2026 Jun 19;17:1861490. doi: 10.3389/fimmu.2026.1861490 (PMC13327901; doi:10.3389/fimmu.2026.1861490)
Supplement: Supplementary file 2 [file DataSheet2.docx]

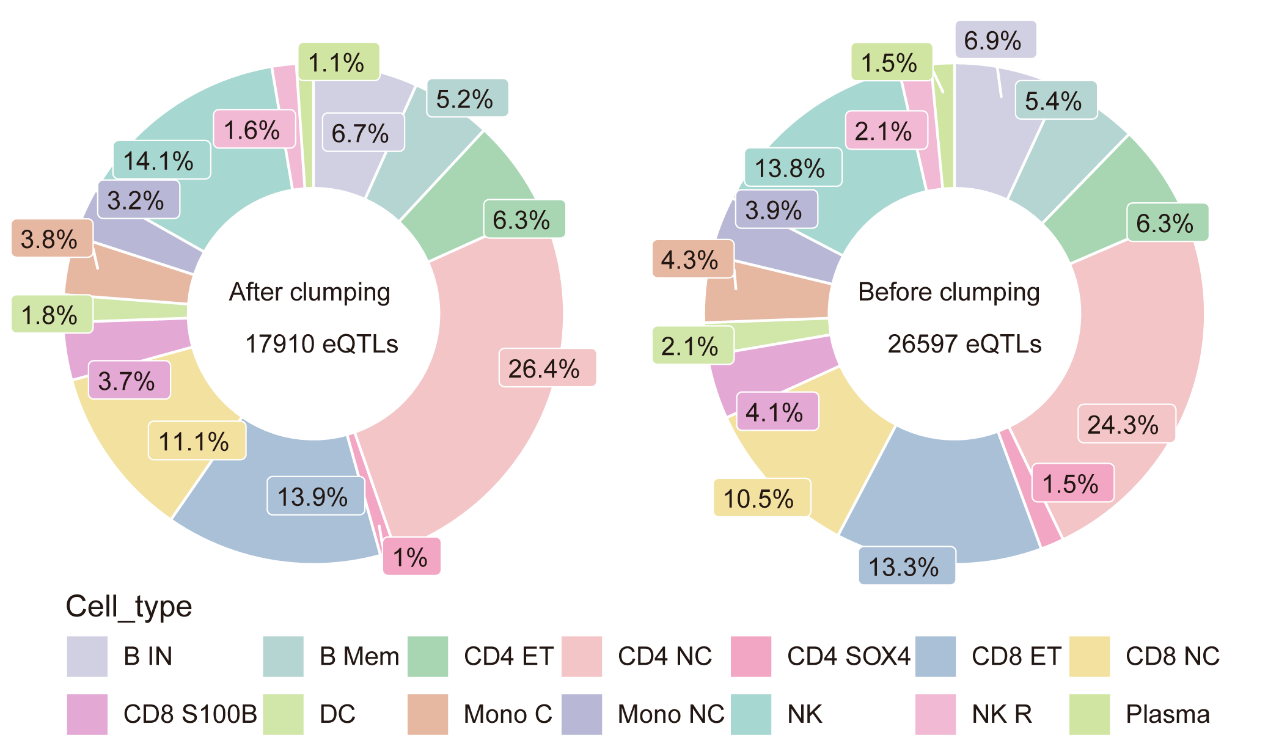


**Figure S1 Cell Proportions of cis-eQTLs Before and After Linkage Disequilibrium (LD) Analysis**

**Note**: CD4_NC: CD4 naive and central memory T cells; CD8_NC: CD8 naive and central memory T cells; CD4_ET: CD4+ T cells with effector memory or central memory phenotype; CD8_ET: CD8+ T cells with effector memory or central memory phenotype; CD4_SOX4: SOX4-expressing CD4+ T cells; CD8_S100B: S100B-expressing CD8+ T cells; NK: natural killer cells; NK R: NK-recruiting cells; B_IN: immature B cells; B_Mem: memory B cells; Mono_C: classical monocytes; Mono_NC: non-classical monocytes; DC: dendritic cells; Plasma: plasma cells.


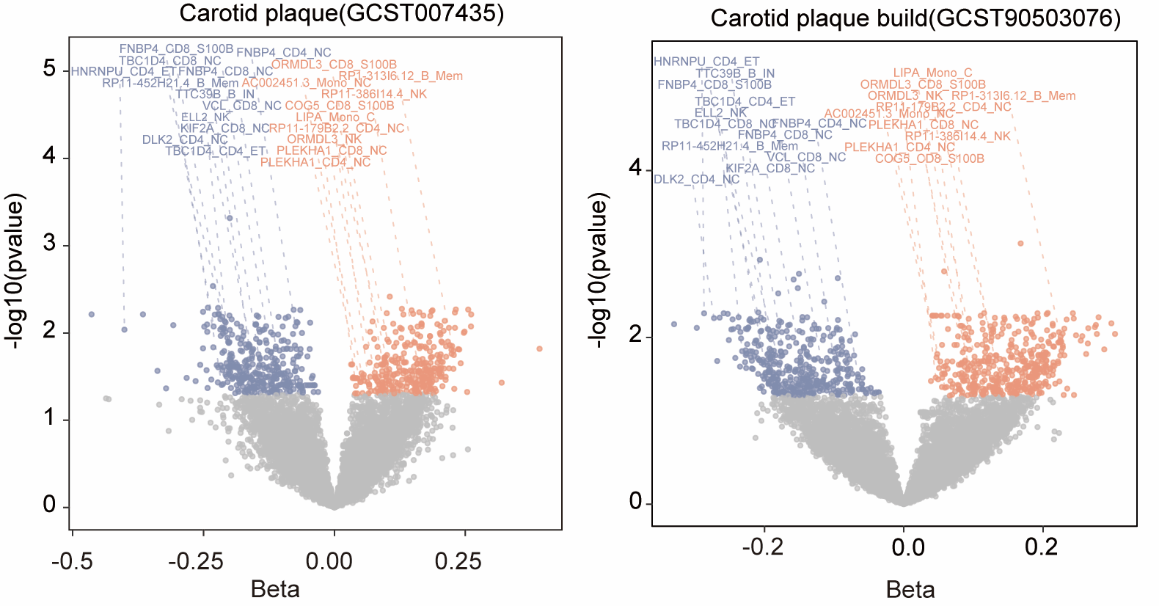


**Figure S2 Volcano Plot of Causal Effects Between Immune Cell-Specific Gene Expression and Carotid Plaque**

**Note**: Orange dots represent immune cell-specific genes that are significantly associated with carotid plaque and have a beta coefficient (β) > 0. Purple dots represent immune cell-specific genes that are significantly associated with carotid plaque and have a beta coefficient (β) < 0. Gray dots represent immune cell-specific genes with no association with carotid plaque. Only immune cell-specific genes that are significantly associated and show consistent directional effects in both outcome datasets are labeled in the plot.


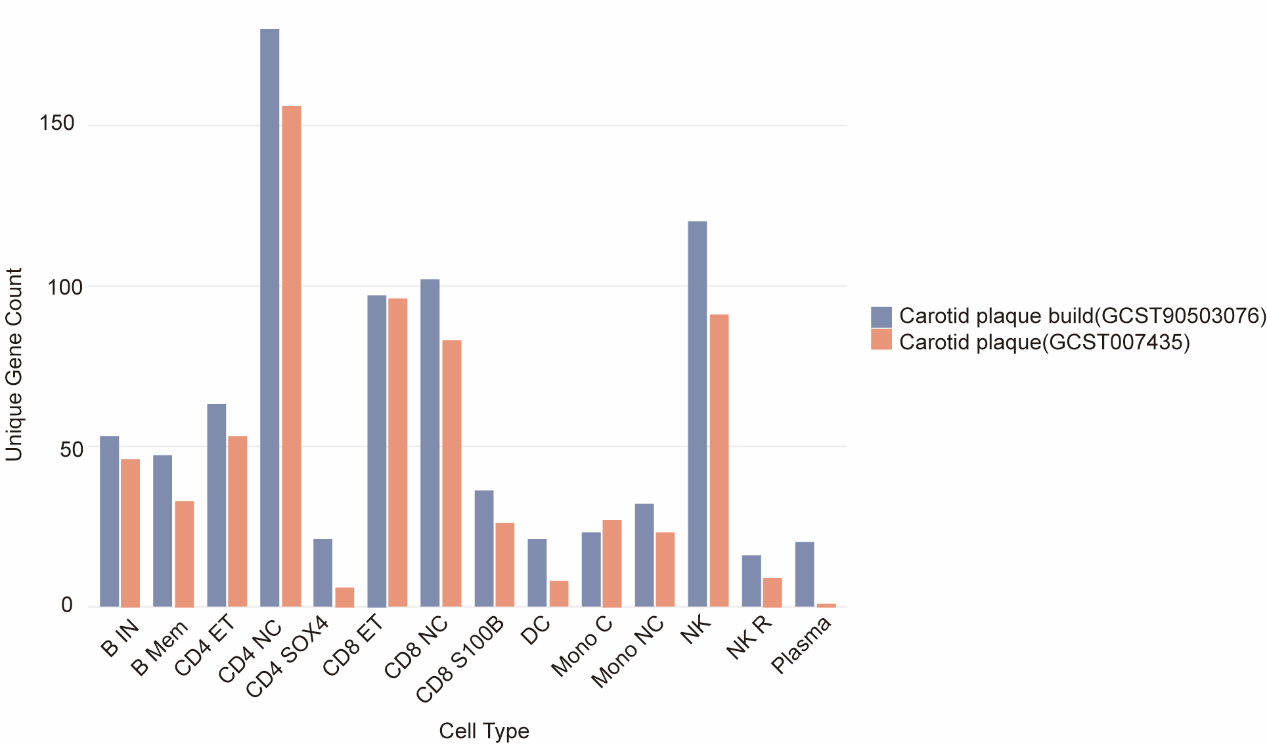


**Figure** **S3 Bar chart of the number of genes and cell types**


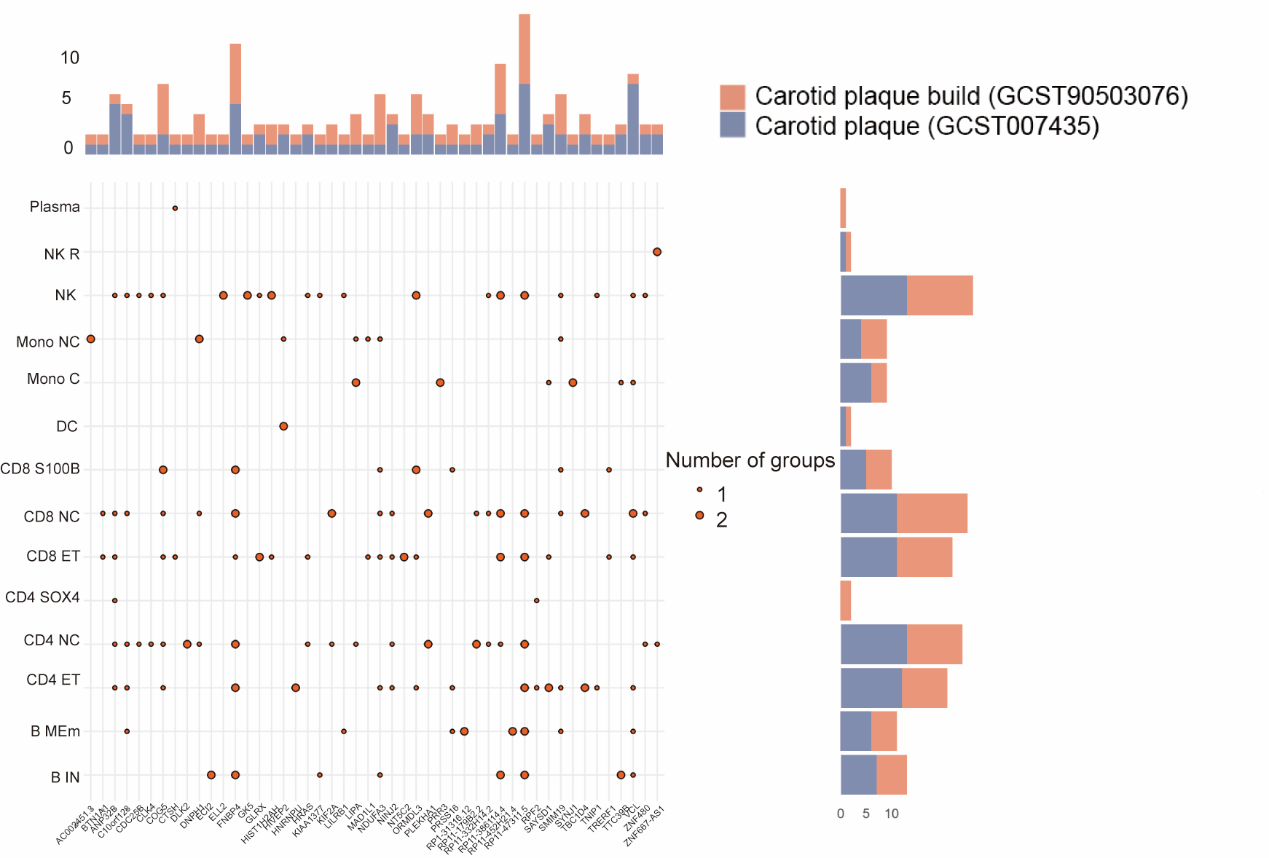


**Figure S4 UpSet plot of MR results**

**Note**: The plot only displays the genes with causal associations with both carotid plaque datasets simultaneously.


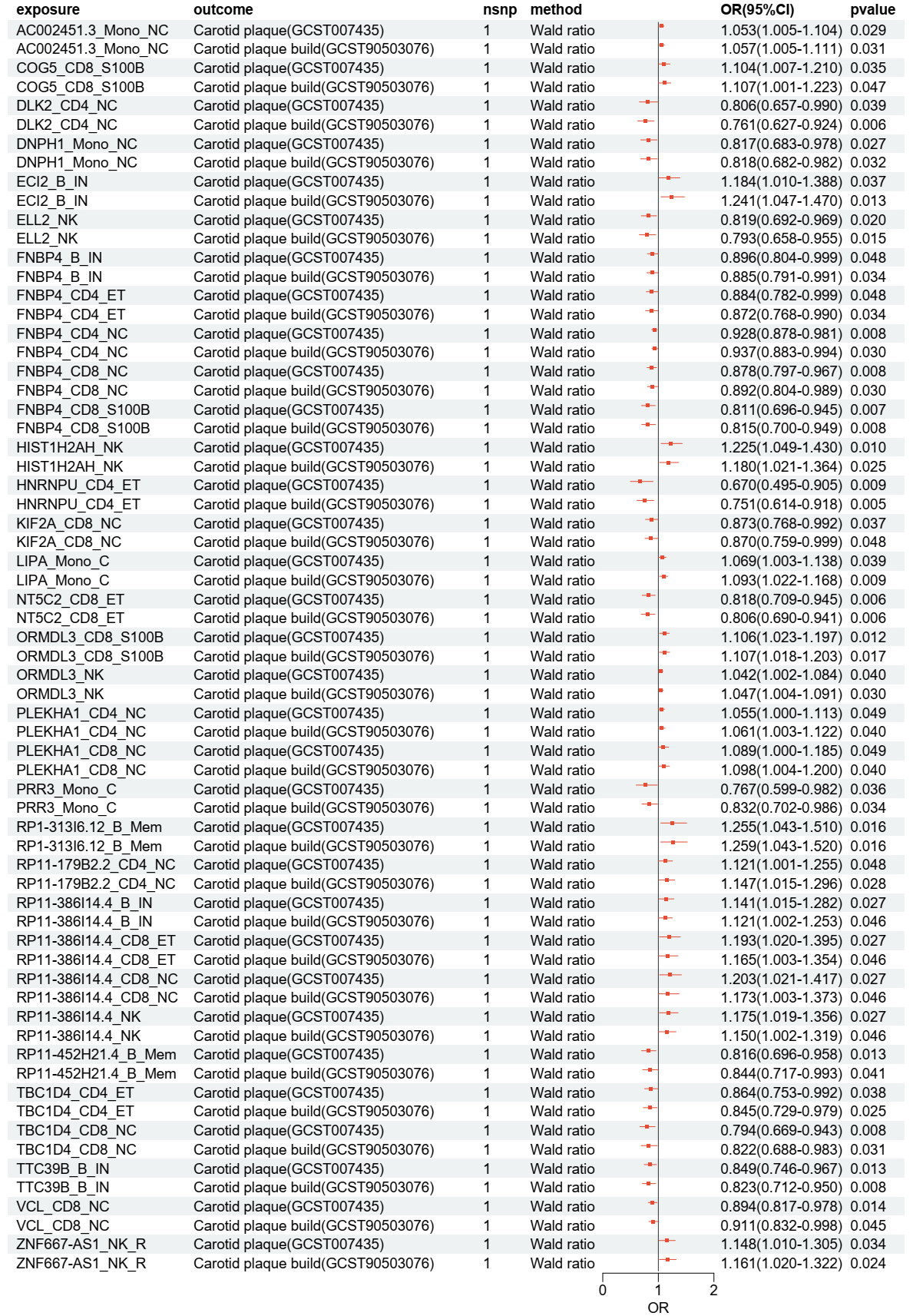
 **Figure S5 MR Causal Association Results Between Immune Cell-Specific Gene Expression and Carotid Plaque**


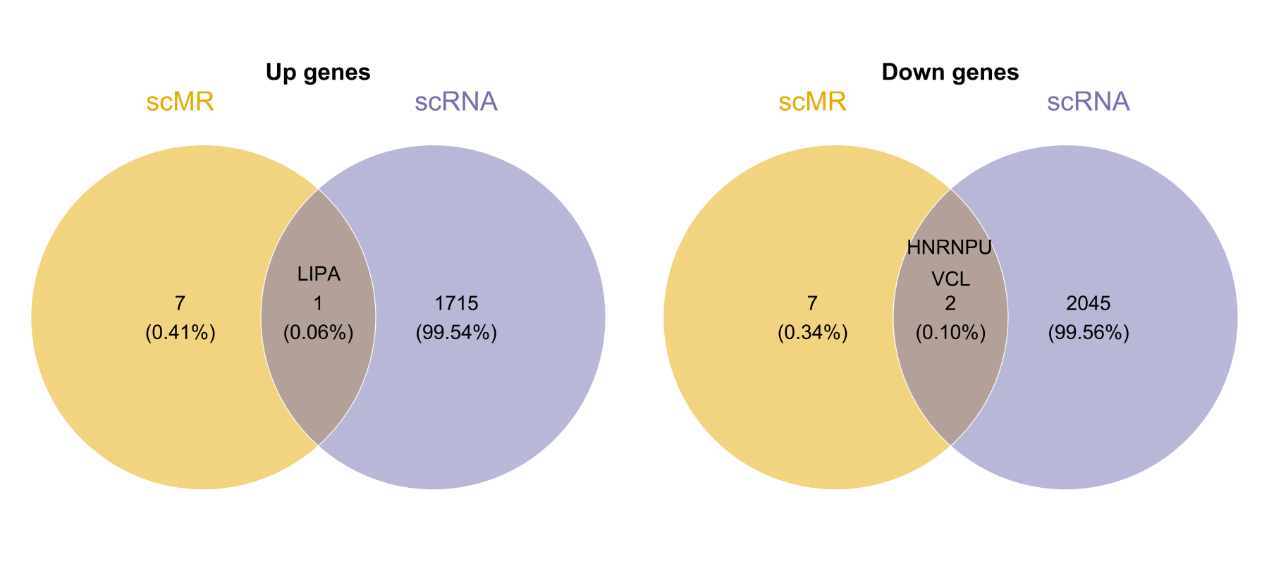
 **Figure S6 Venn Diagram**


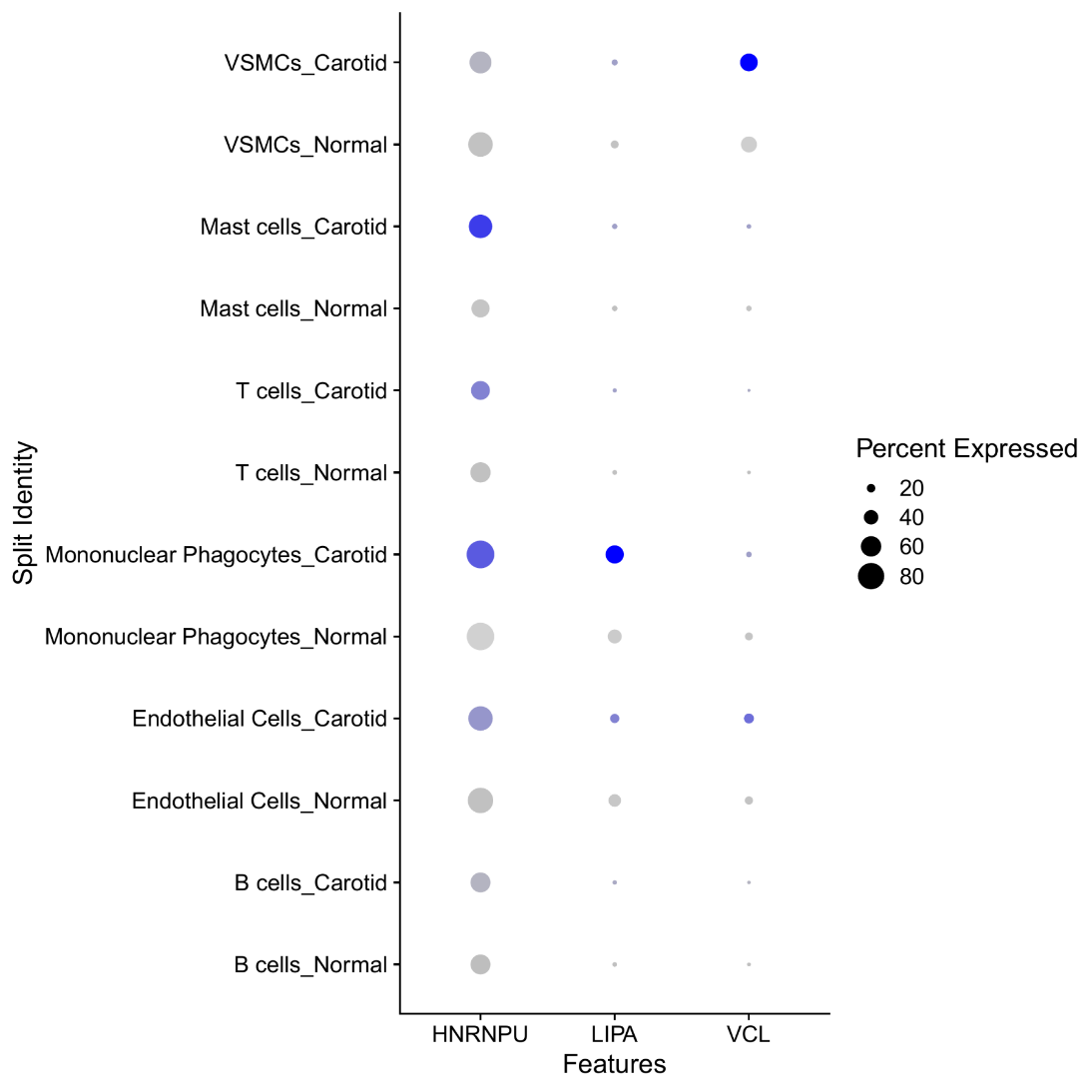


**Figure S7 Dot Plot of Core Gene Expression**

**Note:** Blue indicates the expression level of genes, with the darker the color representing the higher the expression level. The size of the dots represents the proportion of gene expression in cell subsets; the larger the dot, the higher the proportion of gene expression in cells.


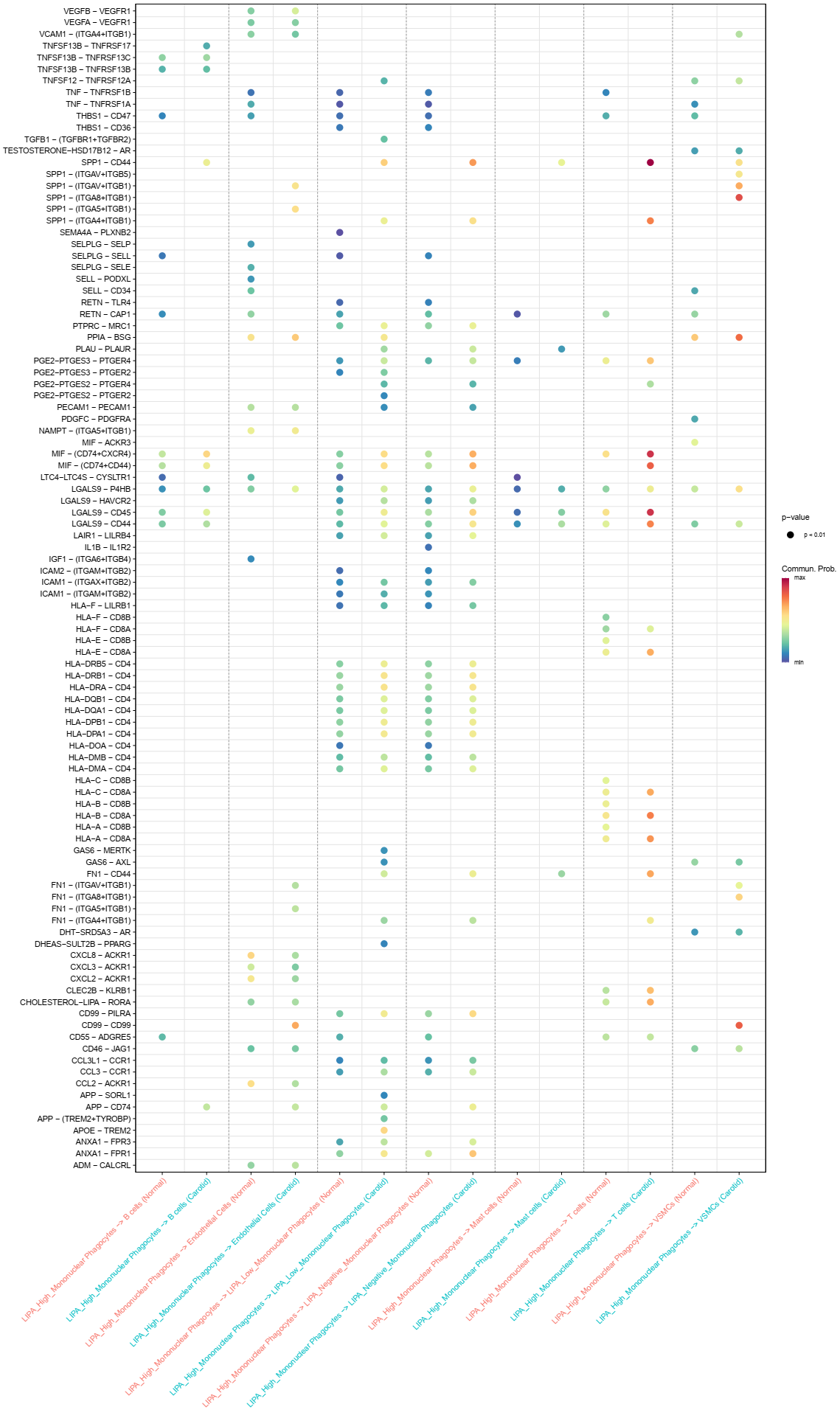


**Figure S8 Cell Interaction Plot of *LIPA* High Mononuclear Phagocytes in the PA and AC Groups**


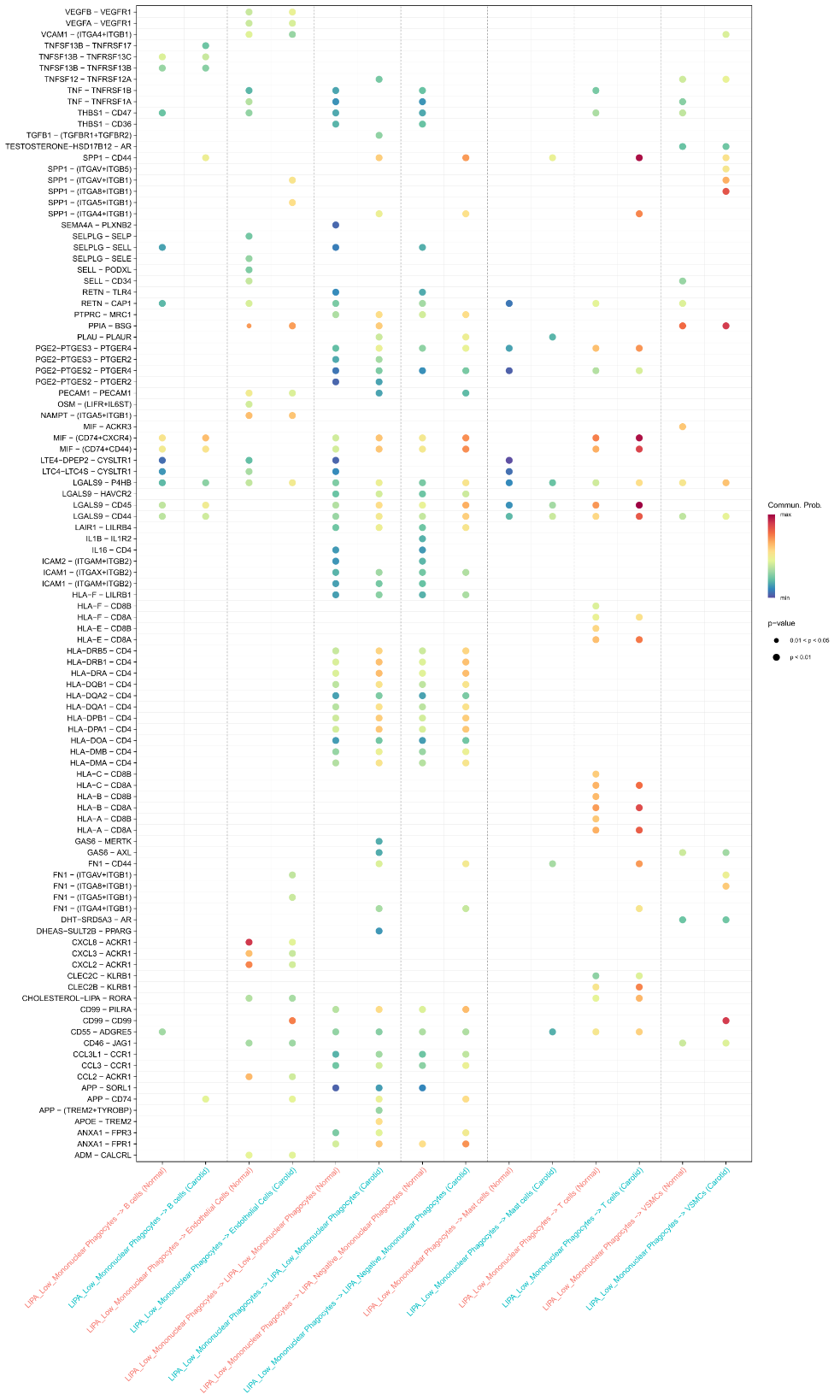


**Figure S9 Cell Interaction Plot of *LIPA* Low Mononuclear Phagocytes in the PA and AC Groups**


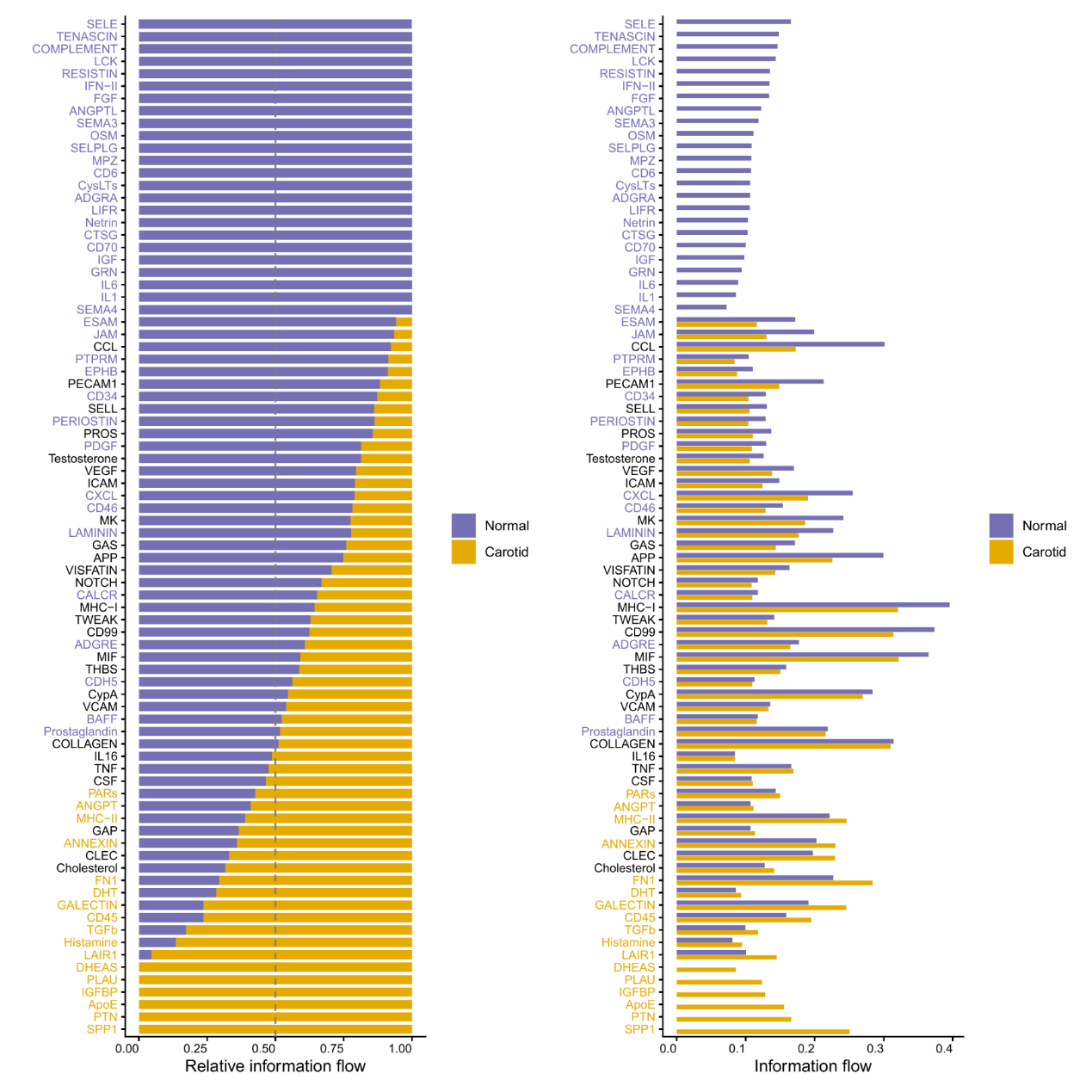


**Figure S10 Bar Plot of Differences in Cell Communication Signaling Pathways Between the PA (Normal) and AC (Carotid) Groups**


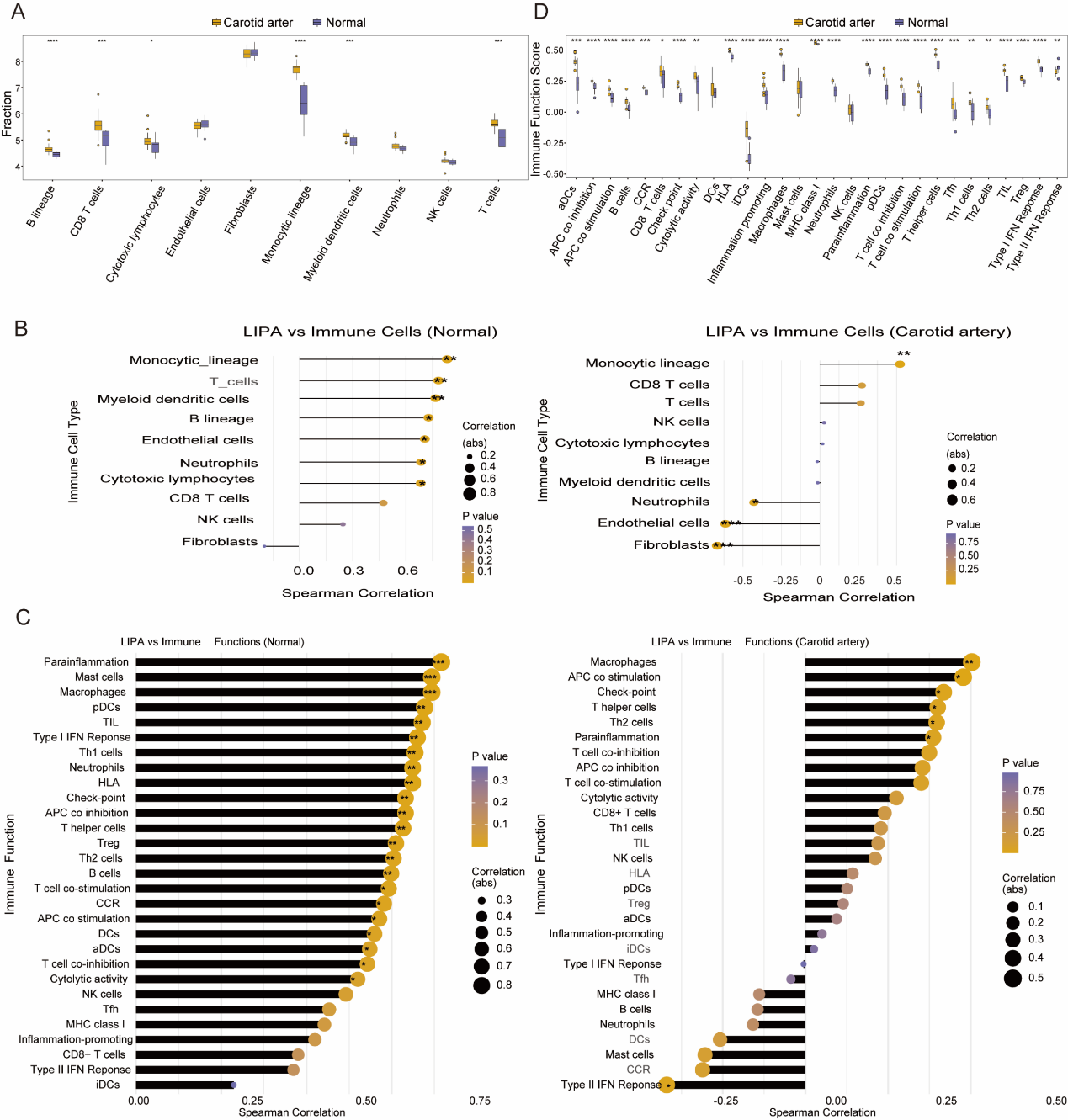


**Figure S11 Evaluation of Immune Infiltration and Immune Function**

**Note:** A. Differential analysis of immune cell infiltration; B. Correlation analysis between *LIPA* expression and immune infiltration; C. Differential analysis of immune function; D. Correlation analysis between *LIPA* expression and immune function.


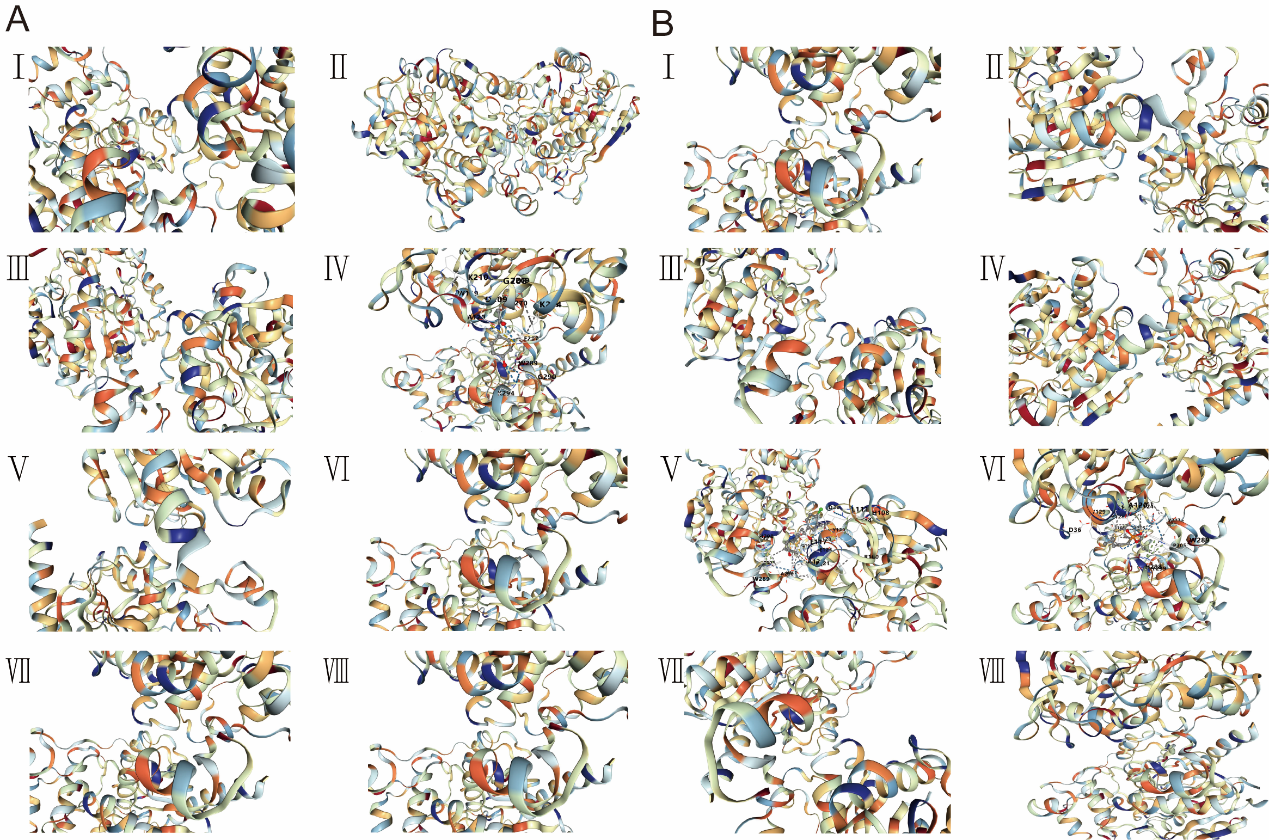


**Figure S12 Molecular Docking Validation**

**Note**: A. Molecular Docking of LIPA: Ⅰ. LIPA docking with (1S)-1-(phenoxymethyl)propyl methylphosphonochloridoate; Ⅱ. LIPA docking with (RP,SP)-O-(2R)-(1-phenoxybut-2-yl)-methylphosphonic acid chloride; Ⅲ. LIPA docking with 4-(5-benzo (1,3) dioxol-5-yl-4-pyridin-2-yl-1H-imidazol-2-yl) benzamide; Ⅳ. LIPA docking with Acetaminophen; Ⅴ. LIPA docking with Benztropine; Ⅵ. LIPA docking with bisphenol A; Ⅶ. LIPA docking with bisphenol AF; Ⅷ. LIPA docking with bisphenol; B. Molecular Docking of LIPA: Ⅰ. LIPA docking with bisphenol F; Ⅱ. LIPA docking with Clozapine; Ⅲ. LIPA docking with Dexamethasone; Ⅳ. LIPA docking with Dorsomorphin; Ⅴ. LIPA docking with Haloperidol; Ⅵ. LIPA docking with hexylphosphonic acid (R)-2-methyl-3-phenylpropyl ester; Ⅶ. LIPA docking with hexylphosphonic acid (S)-2-methyl-3-phenylpropyl ester; Ⅷ. LIPA docking with trichostatin A.


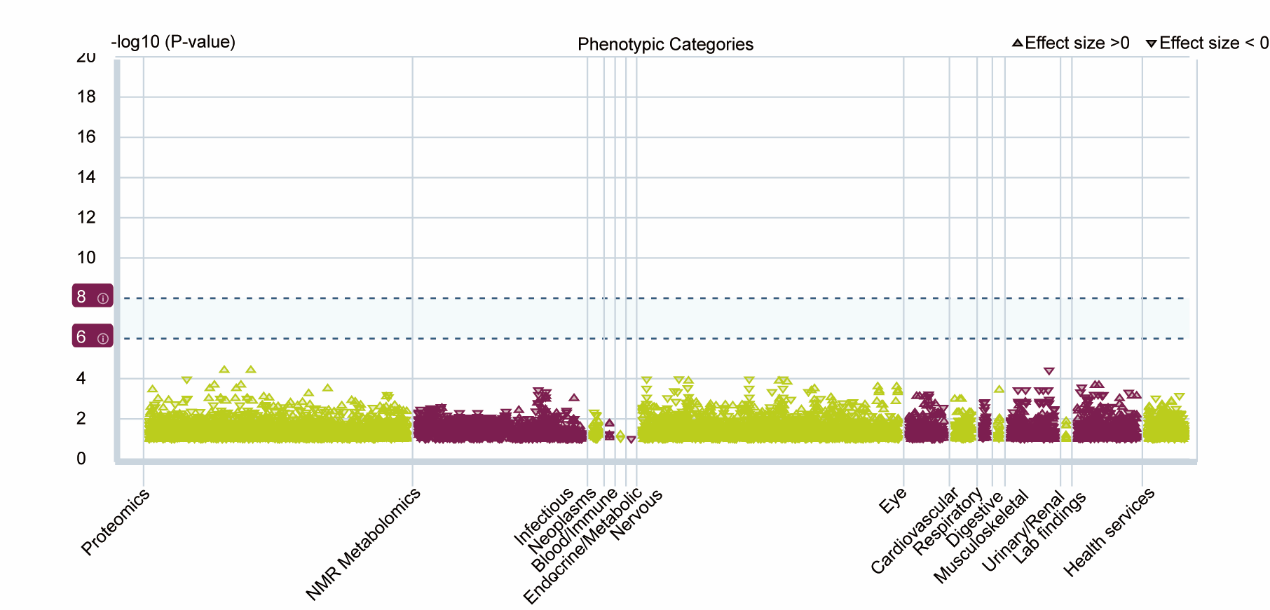


**Figure S13 Association of *LIPA* with Continuous Traits in PheWAS**


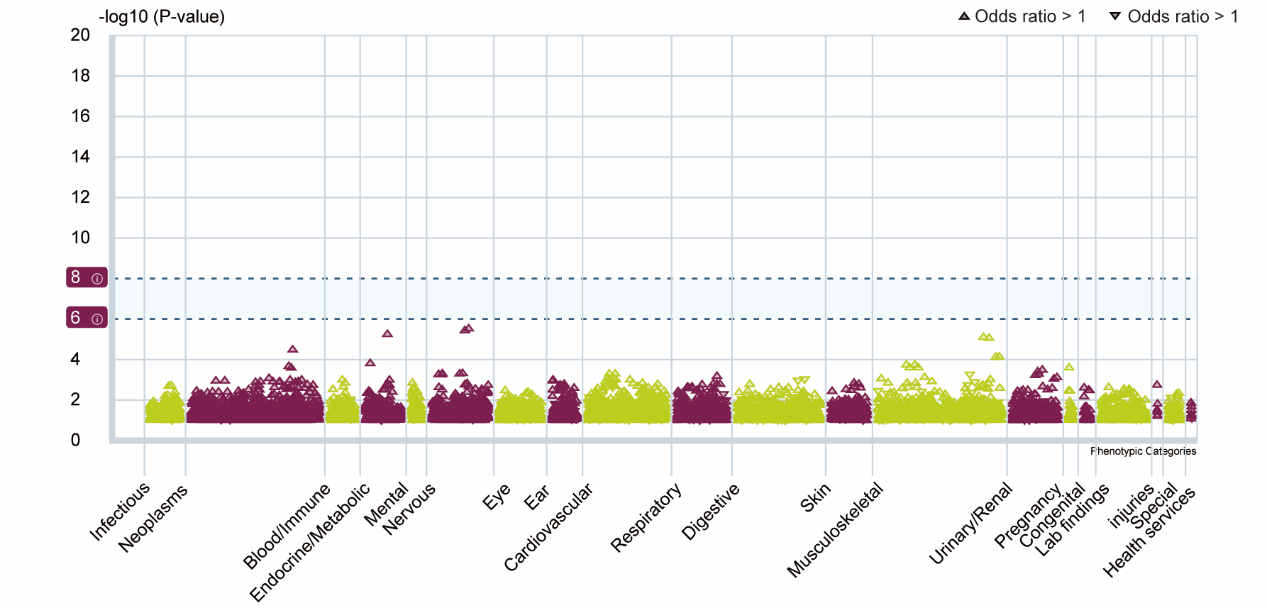


**Figure S14 Association of *LIPA* with Binary Traits in PheWAS**
