# Supplementary material for: Integrative genetic and expression profiling prioritizes LIPA in mononuclear phagocytes as a candidate regulator of carotid plaque
Source: Front Immunol. 2026 Jun 19;17:1861490. doi: 10.3389/fimmu.2026.1861490 (PMC13327901; doi:10.3389/fimmu.2026.1861490)
Supplement: Supplementary file 3 [file Supplementaryfile1.pdf]

frontiers

The International Society for the Advancement of Cytometry (ISAC) has highlighted the importance of including comprehensive methodological information to ensure data reproducibility and reliability. In line with this, Frontiers in Immunology now requires authors to submit a checklist for manuscripts that involve flow or mass cytometry. This checklist helps standardize the reporting process, improving the quality and transparency of published data. By doing so, we support scientific progress, making it easier for other researchers to replicate and validate experiments.

This form should be submitted with any manuscripts using flow or mass cytometry.

Sample/specimen/material description

☐ Total blood

☐ PBMCs

☐ Organ digests

Other Jurkat T cells were purchased from Purcell Life Technology Co., Ltd. in China.

Did the samples suffer any treatment before or after incubation with the antibodies?

☑ Drug Yes. Cells were treated with oxLDL, recombinant SPP1 protein and anti-CD44 antibody.

☐ Cell permeabilization ____________________

☑ Dye Yes. PE-conjugated anti-PD-1 and APC-conjugated anti-LAG-3 fluorescent antibodies were used

☑ Propidium iodine

☐ Not applicable

Other ____________________

Instrument and antibodies

Name of the Cytometer **ATTUNE NXT, A24858**

**Antibodies and targets**	**Fluorochrome/ Metal**	**Catalog number/Company**
LAG-3	FITC	AB_2572484/ ThermoFisher Scientific

PL-1	FITC	HB613117/ abinScience
Anti-CD44	-	ab157107/abcam

Data analyses

1. Name of the software_ FlowJo_v10.8.1 /Modfit__
2. Reference gating strategy in the manuscript or supplementary material Gating strategy in (eg Figure X) _A horizontal axis greater than or equal to 10^3 is considered positive._
